# Cost-effectiveness of a novel AI technology to quantify coronary inflammation and cardiovascular risk in patients undergoing routine coronary computed tomography angiography

**DOI:** 10.1093/ehjqcco/qcae085

**Published:** 2024-09-28

**Authors:** Apostolos Tsiachristas, Kenneth Chan, Elizabeth Wahome, Ben Kearns, Parijat Patel, Maria Lyasheva, Nigar Syed, Sam Fry, Thomas Halborg, Henry West, Edward Nicol, David Adlam, Bhavik Modi, Attila Kardos, John P Greenwood, Nikant Sabharwal, Giovanni Luigi De Maria, Shahzad Munir, Elisa McAlindon, Yogesh Sohan, Pete Tomlins, Muhammad Siddique, Cheerag Shirodaria, Ron Blankstein, Milind Desai, Stefan Neubauer, Keith M Channon, John Deanfield, Ron Akehurst, Charalambos Antoniades, the ORFAN Consortium, Sheena Thomas, Jon Denton, Robyn Farrall, Caroline Taylor, Wendy Qin, Mary Kasongo, Chrisha Ledesma, Damaris Darby, Bruno Silva Santos, Alexios S Antonopoulos, Michail C Mavrogiannis, Andrew Kelion, Susan Anthony, Adrian Banning, Cheng Xie, Rafail A Kotronias, Lucy Kingham, Rajesh K Kharbanda, Chris Mathers, Tarun K Mittal, Anne Rose, George Hudson, Amrita Bajaj, Intrajeet Das, Aparna Deshpande, Praveen Rao, Dan Lawday, Francesca Pugliese, Steffen E Petersen, Saeed Mirsadraee, Nicholas Screaton, Jonathan Rodrigues, Benjamin Hudson, John Graby, Colin Berry, Mohamed Marwan, Pál Maurovich-Horvat, Guo-Wei He, Wen-Hua Lin, Li-Juan Fan, Naohiko Takahashi, Hidekazu Kondo, Neng Dai, Junbo Ge, Bon-Kwon Koo, Marco Guglielmo, Gianluca Pontone, Daniel Huck, Theodora Benedek, Ronak Rajani, Dijana Vilic, Haleema Aljazzaf, Mak S Mun, Giulia Benedetti, Rebecca L Preston, Zahra Raisi-Estabragh, Derek L Connolly, Vinoda Sharma, Rebecca Grenfell, William Bradlow, Matthias Schmitt, Fabiano Serfaty, Ilan Gottlieb, Mario F T Neves, David E Newby, Marc R Dweck, Bernard J Gersh, Stéphane Hatem, Alban Redheuil, Georgios Benetos, Meinrad Beer, Gastón A Rodriguez-Granillo, Joseph Selvanayagam, Francisco Lopez-Jimenez, Ruben De Bosscher, Alain Tavildari, Gemma Figtree, Ibrahim Danad, Ronney Shantouf, Bas Kietselaer, Dimitris Tousoulis, George Dangas, Nehal N Mehta, Christos Kotanidis, Vijay Kunadian, Timothy A Fairbairn

**Affiliations:** Nuffield Department of Primary Care Health Sciences & Department of Psychiatry, University of Oxford, Oxford, OX2 6GG, UK; Acute Multidisciplinary Imaging & Interventional Centre, British Heart Foundation (BHF) Centre of Research Excellence, Division of Cardiovascular Medicine, Radcliffe Department of Medicine, Oxford NIHR Biomedical Research Centre, University of Oxford, John Radcliffe Hospital, Headley Way, Oxford, OX3 9DU, UK; Acute Multidisciplinary Imaging & Interventional Centre, British Heart Foundation (BHF) Centre of Research Excellence, Division of Cardiovascular Medicine, Radcliffe Department of Medicine, Oxford NIHR Biomedical Research Centre, University of Oxford, John Radcliffe Hospital, Headley Way, Oxford, OX3 9DU, UK; Lumanity, Sheffield, S1 2GQ, UK; Acute Multidisciplinary Imaging & Interventional Centre, British Heart Foundation (BHF) Centre of Research Excellence, Division of Cardiovascular Medicine, Radcliffe Department of Medicine, Oxford NIHR Biomedical Research Centre, University of Oxford, John Radcliffe Hospital, Headley Way, Oxford, OX3 9DU, UK; Acute Multidisciplinary Imaging & Interventional Centre, British Heart Foundation (BHF) Centre of Research Excellence, Division of Cardiovascular Medicine, Radcliffe Department of Medicine, Oxford NIHR Biomedical Research Centre, University of Oxford, John Radcliffe Hospital, Headley Way, Oxford, OX3 9DU, UK; Caristo Diagnostics, Oxford, OX2 0HP, UK; Caristo Diagnostics, Oxford, OX2 0HP, UK; Acute Multidisciplinary Imaging & Interventional Centre, British Heart Foundation (BHF) Centre of Research Excellence, Division of Cardiovascular Medicine, Radcliffe Department of Medicine, Oxford NIHR Biomedical Research Centre, University of Oxford, John Radcliffe Hospital, Headley Way, Oxford, OX3 9DU, UK; Acute Multidisciplinary Imaging & Interventional Centre, British Heart Foundation (BHF) Centre of Research Excellence, Division of Cardiovascular Medicine, Radcliffe Department of Medicine, Oxford NIHR Biomedical Research Centre, University of Oxford, John Radcliffe Hospital, Headley Way, Oxford, OX3 9DU, UK; Sydney Medical School, University of Sydney, Sydney, Camperdown, NSW 2050, Australia; Departments of Cardiology and Radiology, Royal Brompton Hospital, London, SW3 6NP, UK; School of Biomedical Engineering and Imaging Sciences, King's College, London, SE1 7EH, UK; Department of Cardiovascular Sciences, University of Leicester, Leicester, LE1 7RH, UK; NIHR Leicester Biomedical Research Centre, Leicester, LE3 9QP, UK; Department of Cardiovascular Sciences, University of Leicester, Leicester, LE1 7RH, UK; NIHR Leicester Biomedical Research Centre, Leicester, LE3 9QP, UK; Department of Cardiology, Translational Cardiovascular Research Group, Milton Keynes University Hospital NHS Foundation Trust, Milton, MK6 5LD, UK; Leeds Teaching Hospitals, Leeds, LS1 3EX, UK; Baker Heart and Diabetes Institute, Melbourne, Victoria, 3004, Australia; Acute Multidisciplinary Imaging & Interventional Centre, British Heart Foundation (BHF) Centre of Research Excellence, Division of Cardiovascular Medicine, Radcliffe Department of Medicine, Oxford NIHR Biomedical Research Centre, University of Oxford, John Radcliffe Hospital, Headley Way, Oxford, OX3 9DU, UK; Acute Multidisciplinary Imaging & Interventional Centre, British Heart Foundation (BHF) Centre of Research Excellence, Division of Cardiovascular Medicine, Radcliffe Department of Medicine, Oxford NIHR Biomedical Research Centre, University of Oxford, John Radcliffe Hospital, Headley Way, Oxford, OX3 9DU, UK; Royal Wolverhampton NHS Trust, Wolverhampton, WV10 0QP, UK; Royal Wolverhampton NHS Trust, Wolverhampton, WV10 0QP, UK; Caristo Diagnostics, Oxford, OX2 0HP, UK; Caristo Diagnostics, Oxford, OX2 0HP, UK; Caristo Diagnostics, Oxford, OX2 0HP, UK; Acute Multidisciplinary Imaging & Interventional Centre, British Heart Foundation (BHF) Centre of Research Excellence, Division of Cardiovascular Medicine, Radcliffe Department of Medicine, Oxford NIHR Biomedical Research Centre, University of Oxford, John Radcliffe Hospital, Headley Way, Oxford, OX3 9DU, UK; Caristo Diagnostics, Oxford, OX2 0HP, UK; Brigham and Women's Hospital, Harvard Medical School, Boston, MA, 02115, USA; Department of Cardiovascular Medicine, Cleveland Clinic Heart Vascular and Thoracic Institute, Cleveland, OH, 44195, USA; Acute Multidisciplinary Imaging & Interventional Centre, British Heart Foundation (BHF) Centre of Research Excellence, Division of Cardiovascular Medicine, Radcliffe Department of Medicine, Oxford NIHR Biomedical Research Centre, University of Oxford, John Radcliffe Hospital, Headley Way, Oxford, OX3 9DU, UK; Acute Multidisciplinary Imaging & Interventional Centre, British Heart Foundation (BHF) Centre of Research Excellence, Division of Cardiovascular Medicine, Radcliffe Department of Medicine, Oxford NIHR Biomedical Research Centre, University of Oxford, John Radcliffe Hospital, Headley Way, Oxford, OX3 9DU, UK; Institute of Cardiovascular Science, University College London, London, WC1E 6DD, UK; Lumanity, Sheffield, S1 2GQ, UK; Acute Multidisciplinary Imaging & Interventional Centre, British Heart Foundation (BHF) Centre of Research Excellence, Division of Cardiovascular Medicine, Radcliffe Department of Medicine, Oxford NIHR Biomedical Research Centre, University of Oxford, John Radcliffe Hospital, Headley Way, Oxford, OX3 9DU, UK

**Keywords:** Cost-effectiveness analysis, Coronary artery disease, Inflammation, Coronary CT angiography

## Abstract

**Aims:**

Coronary computed tomography angiography (CCTA) is a first-line investigation for chest pain in patients with suspected obstructive coronary artery disease (CAD). However, many acute cardiac events occur in the absence of obstructive CAD. We assessed the lifetime cost-effectiveness of integrating a novel artificial intelligence-enhanced image analysis algorithm (AI-Risk) that stratifies the risk of cardiac events by quantifying coronary inflammation, combined with the extent of coronary artery plaque and clinical risk factors, by analysing images from routine CCTA.

**Methods and results:**

A hybrid decision-tree with population cohort Markov model was developed from 3393 consecutive patients who underwent routine CCTA for suspected obstructive CAD and followed up for major adverse cardiac events over a median (interquartile range) of 7.7(6.4–9.1) years. In a prospective real-world evaluation survey of 744 consecutive patients undergoing CCTA for chest pain investigation, the availability of AI-Risk assessment led to treatment initiation or intensification in 45% of patients. In a further prospective study of 1214 consecutive patients with extensive guidelines recommended cardiovascular risk profiling, AI-Risk stratification led to treatment initiation or intensification in 39% of patients beyond the current clinical guideline recommendations. Treatment guided by AI-Risk modelled over a lifetime horizon could lead to fewer cardiac events (relative reductions of 11%, 4%, 4%, and 12% for myocardial infarction, ischaemic stroke, heart failure, and cardiac death, respectively). Implementing AI-Risk Classification in routine interpretation of CCTA is highly likely to be cost-effective (incremental cost-effectiveness ratio £1371–3244), both in scenarios of current guideline compliance, or when applied only to patients without obstructive CAD.

**Conclusions:**

Compared with standard care, the addition of AI-Risk assessment in routine CCTA interpretation is cost-effective, by refining risk-guided medical management.

## Graphical Abstract

**Graphical Abstract:**
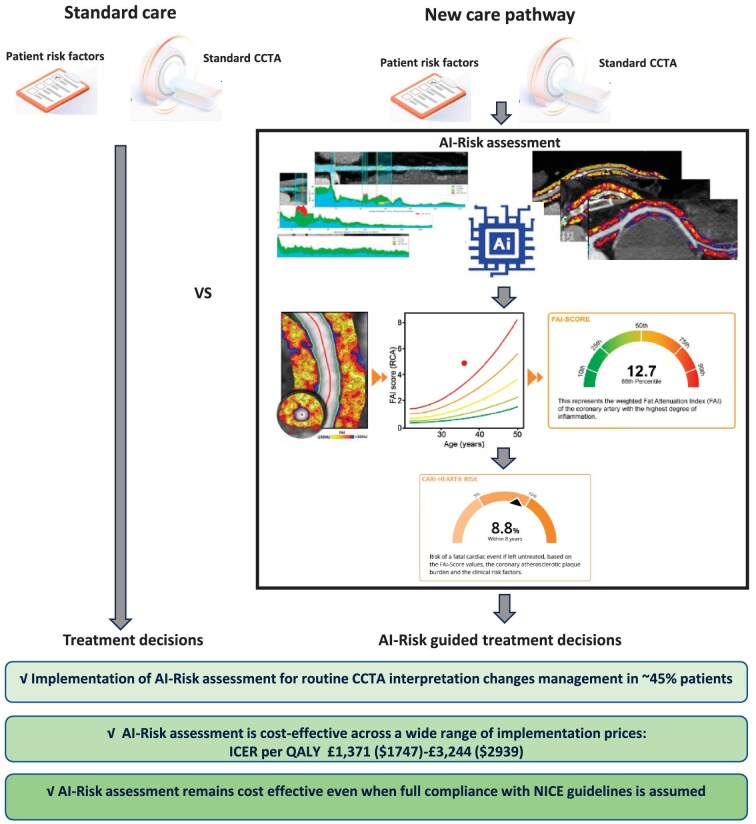

Key learning points **What is already known**

- Coronary computed tomography angiography (CCTA) is the first-line investigation for chest pain, but only approximately 20% scans demonstrated obstructive coronary artery disease (CAD).
- Coronary inflammation could be assessed non-invasively on routine acquired CCTA, and integrated with clinical risk factors using artificial intelligence (AI-Risk) that predicts future cardiac events even in the absence of obstructive CAD.

**What this study adds**

- Implementation of AI-Risk Classification in the UK National Health Service for the analysis of CCTA scans, performed as part of standard care for investigation of chest pain, leads to changes of management in up to 45% of patients.
- Integration of AI-Risk Classification in routine CCTA interpretation is highly likely to represent value for money across a range of implementation price points over a lifetime horizon.
- AI-Risk assessment remains cost-effective even when full compliance with National Institute for Health and Care Excellence guidelines is assumed.

## Introduction

Cardiovascular disease (CVD) is a leading cause of death worldwide and is associated with significant socioeconomic burden.^1^ Contemporary CVD risk assessment in the general population is currently based on demographic characteristics and clinical risk, while the 10-year risk of fatal and non-fatal CVD is then calculated using risk scores such as QRISK3,^2^ SCORE2,^3^ or others, to guide preventive treatments (e.g. statin treatment if CVD risk ≥10%). The increasing number of individuals undergoing coronary computed tomography angiography (CCTA) as first-line investigation for suspected coronary artery disease (CAD) offers a unique opportunity to refine cardiovascular risk management, moving from the use of clinical factors-based risk calculators (e.g. QRISK3) to a more refined risk classification system taking into account information about coronary atherosclerosis and disease activity, extractable from routine CCTA.^4^

A new artificial intelligence-enhanced cardiac risk prediction model (AI-Risk model) has been developed by combining clinical risk factors with quantitative metrics from routine CCTA, including coronary inflammation measured by perivascular fat attenuation index (FAI) score and the extent of coronary atherosclerosis,^5,6^ to provide individualized cardiac risk prediction. This model has been incorporated into a regulatory cleared medical device (CaRi-Heart^®^, Caristo Diagnostics), and it has been deployed recently in clinical practice in Europe, UK, and Australia, as a tool to guide initiation or intensification of risk-reduction interventions. A previous study has shown that the new AI-Risk Classification significantly improves prediction of major adverse cardiac events (MACE) among patients undergoing CCTA in the UK (vs. QRISK3),^7^ while statin treatment reduces coronary inflammation and the residual inflammatory risk captured by AI-Risk Classification.^8,9^ This would support the deployment of preventative treatments in these individuals to improve their cardiovascular outcomes, thereby reducing the socioeconomic impact of acute cardiovascular events.

This study aimed to evaluate the cost-effectiveness of incorporating the AI-Risk Classification system within the existing clinical care pathway of stable chest pain, in the context of the UK National Health Service (NHS).

## Methods

### Study design and clinical cohorts

The cost-effectiveness model was based on the input parameters derived from three patient cohorts and a published meta-analysis as follows and the flow-diagram for study design is presented in Supplementary materials online, *Figure S1*:

1. A long-term outcomes cohort study. The Oxford Risk Factor and Non-invasive imaging (ORFAN) study (NCT05169333) that evaluated the prognostic value of AI-Risk Classification against true cardiac events recorded during prospective follow up.^7^
2. A prospective real world evaluation survey. Assessing the impact of the AI-Risk Classification system on medical management after CCTA.
3. A prospective clinical study. Designed to evaluate the reclassification achieved using the AI-Risk Classification system, and to model the impact of AI-Risk deployment on patient management, assuming absolute compliance with current National Institute for Health and Care Excellence (NICE) clinical guidelines.^4^
4. A published meta-analysis of all outcomes clinical trials was used to model the effect size of statin treatment on various cardiovascular outcomes, based on the baseline risk level of the individuals treated.^11^ A brief description of these is presented below, and further details are included in the supplementary materials.

### Long-term outcomes cohort study

**Figure 1:**
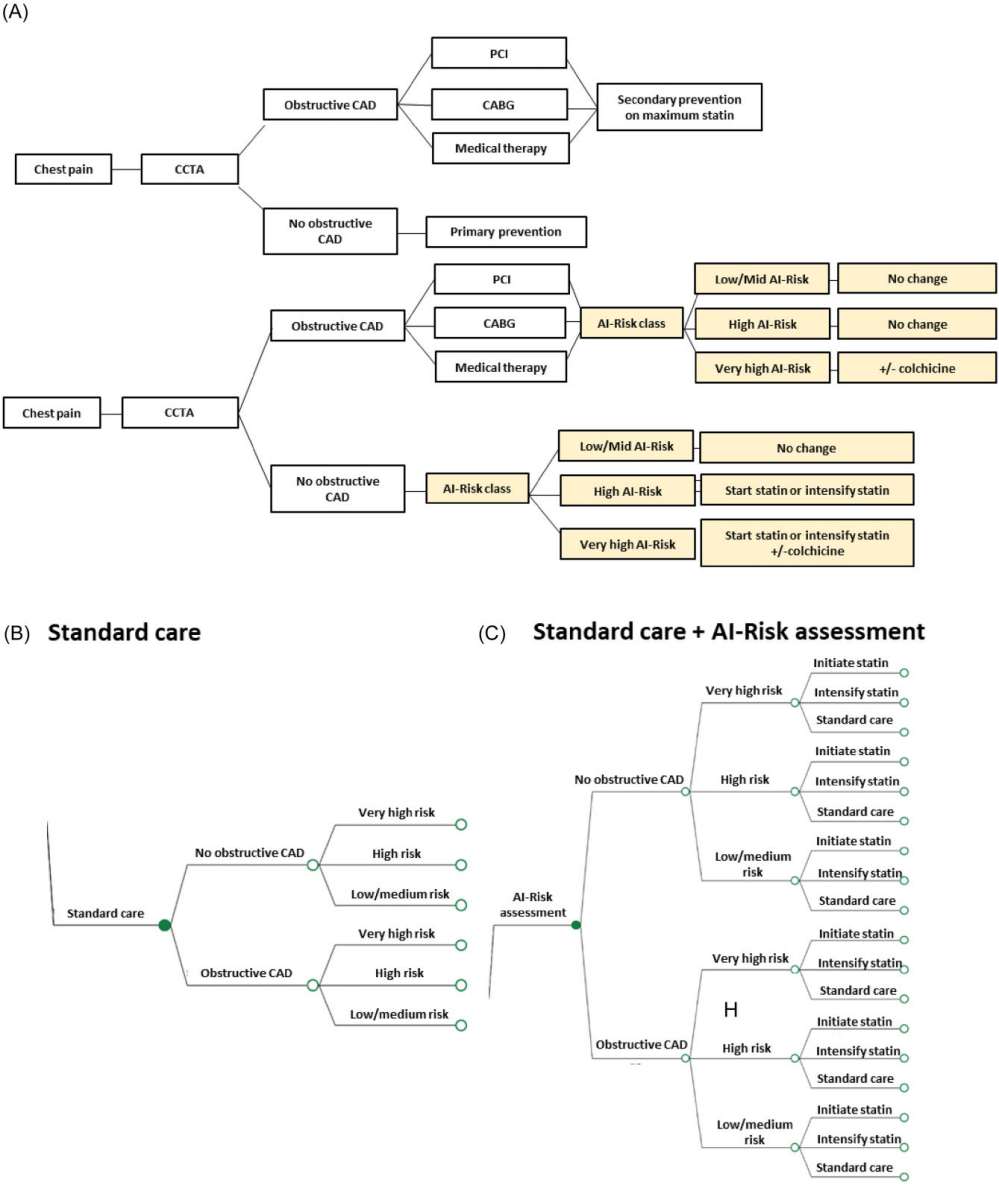
Decision tree for patient undergoing coronary computed tomography angiogram for suspected coronary artery disease. (A) Proposed clinical pathway of additional AI-Risk Classification to standard care. (B) Decision tree for standard care with CCTA assessment of presence/absence of obstructive CAD and clinical risk factor stratification. (C) Decision tree after addition of AI-Risk assessment. CAD, coronary artery disease; CABG, coronary artery bypass grafting; CCTA, coronary computed tomography angiogram; and PCI, percutaneous coronary intervention.

Consecutive patients from the ORFAN study (*n* = 3393) were followed for a median of 7.7-years (interquartile range 6.4–9.1 years) for MACE, including myocardial infarction (MI), ischaemic stroke, heart failure, and cardiac mortality (Detailed definitions are provided in Supplementary materials online, *Table S1*). The presence or absence of obstructive CAD on CCTA was used to define the initial CAD status. In the Markov model, this informed the likelihood of transitioning from one health state to another (or remaining in the same health state). The baseline CAD status was defined by the presence/absence of obstructive coronary atherosclerosis based on the Society of Cardiovascular Computed Tomography (SCCT)/the American College of Cardiology (ACC)/the American College of Radiology (ACR)/the North America Society of Cardiovascular Imaging (NASCI) guideline^12^ (*Figure 1*), followed by further stratification based on AI-Risk Classification levels (low/mid, high, and very high risk categories) as previously defined^6,10^ (Supplementary material online, *Figure S2*). The total number of observed MACE (*n* = 706) were divided by the total number of life months (i.e. the accumulated months of being alive across all patients during the observation period) multiplied by 3 (as a cycle in the Markov model is 3 months). As a result, the transition probabilities were equal between the first and subsequent MACE.

### Prospective real-world evaluation survey to compare AI-Risk guided management vs. standard care

To evaluate the impact of AI-Risk Classification on current clinical management (standard care), a prospective, real-world evaluation survey was performed in four NHS Hospitals that were considered representative of the UK population, as described in the  Supplementary material online, *Figure S1* and *Table S2*. After clinical management was decided and recorded by the local clinical care teams (following standard CCTA interpretation for the presence of obstructive CAD, visual assessment of plaque burden, and risk profiling with QRISK3), the AI-Risk Classification results were provided to the same clinicians who were then asked to complete a survey recording its impact on the patient management plan. Data on statin dose was also collected and the mean dose of those who initiated or intensified statin treatment due to the AI-Risk Classification system was recorded. This was performed for each of the six individual risk levels [i.e. three AI-Risk levels (mid/low, high, and very high) and the presence or absence of obstructive CAD].

### Prospective clinical study to compare AI-Risk guided management vs. full compliance with NICE guidance

The full details of the study design are presented in the Supplementary material online, *Table S2* and *Figure S1*. This is a prospective evaluation of the AI-Risk Classification against a theoretical full compliance with the NICE guidelines for CVD prevention,^4^ that included QRISK3 and extensive risk factor profiling in 1214 consecutive patients recruited in four NHS hospitals. The conventional risk classification was defined using medical management that fully adopts the current NICE guidelines and the results of the CCTA interpreted by the referring clinicians for the presence of obstructive CAD.^2^

### Meta-analysis on the effect size of statin treatment on cardiovascular event rates

The treatment effect of statin therapy on MACE was informed by the largest published meta-analysis addressing this question (data from 170 000 participants in 26 randomized trials, Cholesterol Treatment Trialists’ Collaboration).^11^ The low-density lipoprotein (LDL) cholesterol (mmol/l) reduced by an average of 48% with 40 mg of Atorvastatin and 53% with 80 mg Atorvastatin compared with placebo.^13^ Using a 3.7 mmol/L baseline LDL cholesterol,^14^ we calculated the relative risk of having MACE when statin (Atorvastatin) was initiated (i.e. from 0 to 40 mg) and intensified (i.e. from 40 to 80 mg) by CAD status and different baseline risk level (i.e. <5%, 5% to <10%, ≥10%) (Supplementary material online, *Tables S3* and *S4*). The summary of statin effect on outcomes by risk groups and detailed calculations for the statin effects are presented in Supplementary material online, *Tables S4* and *S5*.

### CCTA image analysis and calculation of AI-Risk Classification

CCTA images were analysed with CaRi-Heart® v2.5 (Caristo Diagnostics) to generate metrics of coronary inflammation FAI-Score for each of the epicardial coronary arteries (left anterior descending, LAD; left circumflex artery, LCx; and right coronary artery, RCA). AI-Risk was then calculated using CaRi-Heart^®^ v2.5, by incorporating FAI score together with clinical risk factors and extent of CAD into a prognostic model, to predict absolute 8-year cardiac mortality risk. Based on the FAI-Score and AI-Risk results, CaRi-Heart^®^ classifies patients into three risk categories (AI-Risk Classification)^10^:

*Low/medium-risk category*: AI-Risk <5% and FAI-Score <75th percentile in the LAD/RCA and <95th percentile in the LCx.

*High-risk category*: AI-Risk 5% to <10% and/or FAI-Score in the LAD/RCA between 75th and 90th percentile and/or FAI score in the LCx >95th percentile.

*Very high-risk category*: AI-Risk ≥10% and/or FAI-Score at LAD/RCA >90th percentile.

### Model type and structure

A decision analytic model was developed to compare the two alternatives (AI-Risk assessment in addition to standard care^4,15,16^ vs. standard care alone) in terms of costs and QALYs over a patients’ lifetime. The model consisted of a decision tree followed by a population cohort Markov Model. Details on healthcare cost and utility measures are presented in the Supplement. The branches in the decision tree modelled separate individuals with or without CAD at three risk categories defined by the AI-Risk model device (low/medium, high, and very high risk category, as described above). The subsequent nodes following the risk classification corresponded to the management of patients with statins. The treatment effect of AI-Risk was modelled in the decision tree as the change in management in statins compared to statin management following standard care. As such, the last nodes of the AI-Risk model branch were either statin initiation, increased statin dose, or standard care (i.e. statin management did not change with the AI-Risk model). The proposed clinical pathway and decision tree is illustrated in *Figure 1*.

**Figure 2:**
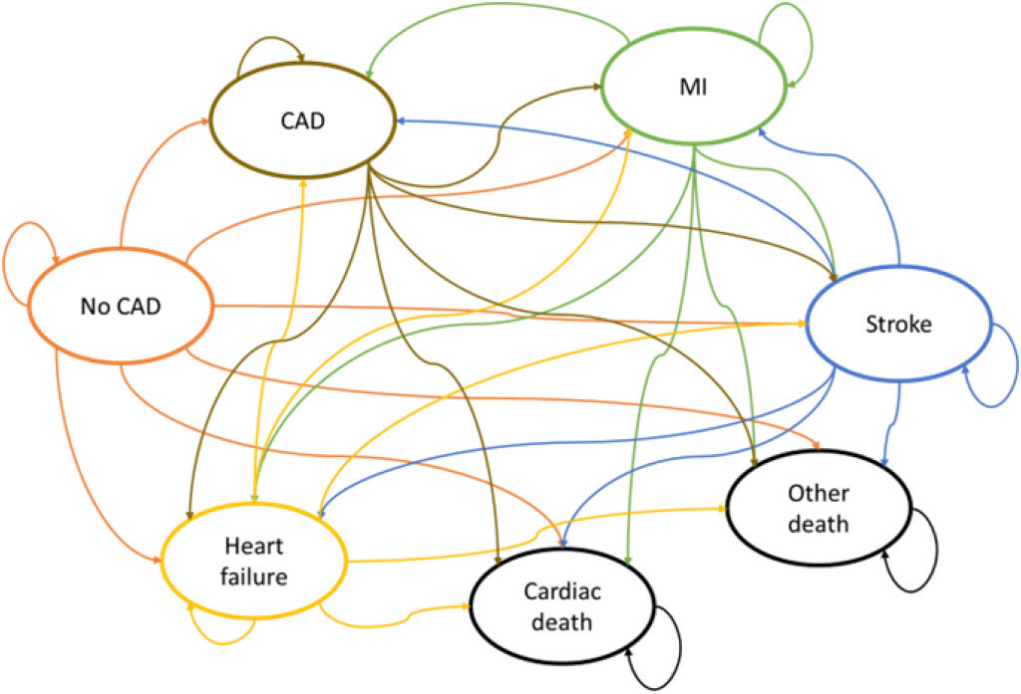
Markov model illustrating transition of health state every 3 months cycle.

Following the decision tree, individuals entered a Markov model health state of no obstructive CAD, MI, ischaemic stroke, heart failure, obstructive CAD, cardiac death, or other death. The initial health state for individuals without existing obstructive CAD was no obstructive CAD. For individuals with existing obstructive CAD, the no obstructive CAD health state was dropped from the Markov model and the initial health state for these individuals was CAD. The Markov model had a cycle of 3 months (individuals could stay in the same health state or transit to other health states once every 3 months) and a 30-year lifetime horizon. The structure of the Markov model is illustrated in *Figure 2*. The economic evaluation used an NHS and personal social services costing perspective, and a discount rate of 3.5% was used for both costs and outcomes. The Health Economics Analysis Plan is presented in the supplementary materials.

### Sensitivity analyses

Several sensitivity analyses were performed, and the parameters used are presented in Supplementary material online, *Table S3*. The scenarios were as follows. (1) Risk category reclassification data from full implementation of NICE guidelines (from the prospective clinical study) instead of real-world evaluation (real world evaluation survey) were used. (2) Treatment management that includes the addition of colchicine (or similar agent/novel therapeutic) to the statin management of a patient at high AI-Risk category with and without obstructive CAD was applied. These data were derived from the prospective real-world evaluation, where initiation of colchicine or other agents was decided by the clinical care teams and recorded for the purpose of this evaluation. In this sensitivity analysis, all patients at very high risk that had initiated or intensified statin treatment due to AI-Risk Classification were assumed to receive colchicine as well. The protective effect of colchicine in very-high risk individuals was assumed to be 0.80, i.e. an additional 20% reduction on the risk of MACE beyond statin treatment based on the recent clinical trial (LoDoCo2)^17^(Supplementary material online, *Table S3*). (3) Patients with existing obstructive CAD were excluded from the analysis to explore the cost-effectiveness of AI-Risk Classification if it were implemented to individuals with no or non-obstructive CAD. (4) The statin effect on MACE was assumed to be halved (50% lower). This might be the case because of reduced clinical adherence and patient compliance to statin therapy, and (5) risk reclassification due to the AI-Risk Classification was reduced by 50% compared with the main analysis, reflecting the possibility that the risk evidence would not necessarily be acted upon as often as was found in our study. These last two scenarios modelled extreme reductions in efficacy/reclassification that are unlikely to be realistic. They were included to stress-test the cost-effectiveness results under highly pessimistic assumptions.

Finally, to express the uncertainty around the estimated incremental cost-effectiveness ratio (ICER; the ratio of incremental costs to incremental QALYs), a probabilistic sensitivity analysis was performed by sampling 1000 sets of input parameters from their pre-specified distributions, resulting in 1000 pairs of estimated incremental costs and incremental outcomes. These simulated ICERs were plotted on cost-effectiveness planes to display uncertainty in the estimated ICER. In addition, a cost-effectiveness acceptability curve (CEAC) was drawn to display the probability of each intervention to be cost-effective at different thresholds for approval.

Cost-effectiveness analyses were conducted using the TreeAge Pro 2019, R2 (TreeAge Software, Williamstown, MA, USA). Descriptive and clinical outcome data analyses were performed using STATA 18.0 (StataCorp LP, College Station, TX, USA). Reporting of the study followed the Consolidated Health Economic Evaluation Reporting Standards (CHEERS) statement in the supplementary materials.^18^

## Results

### Base case analysis

**Figure 3:**
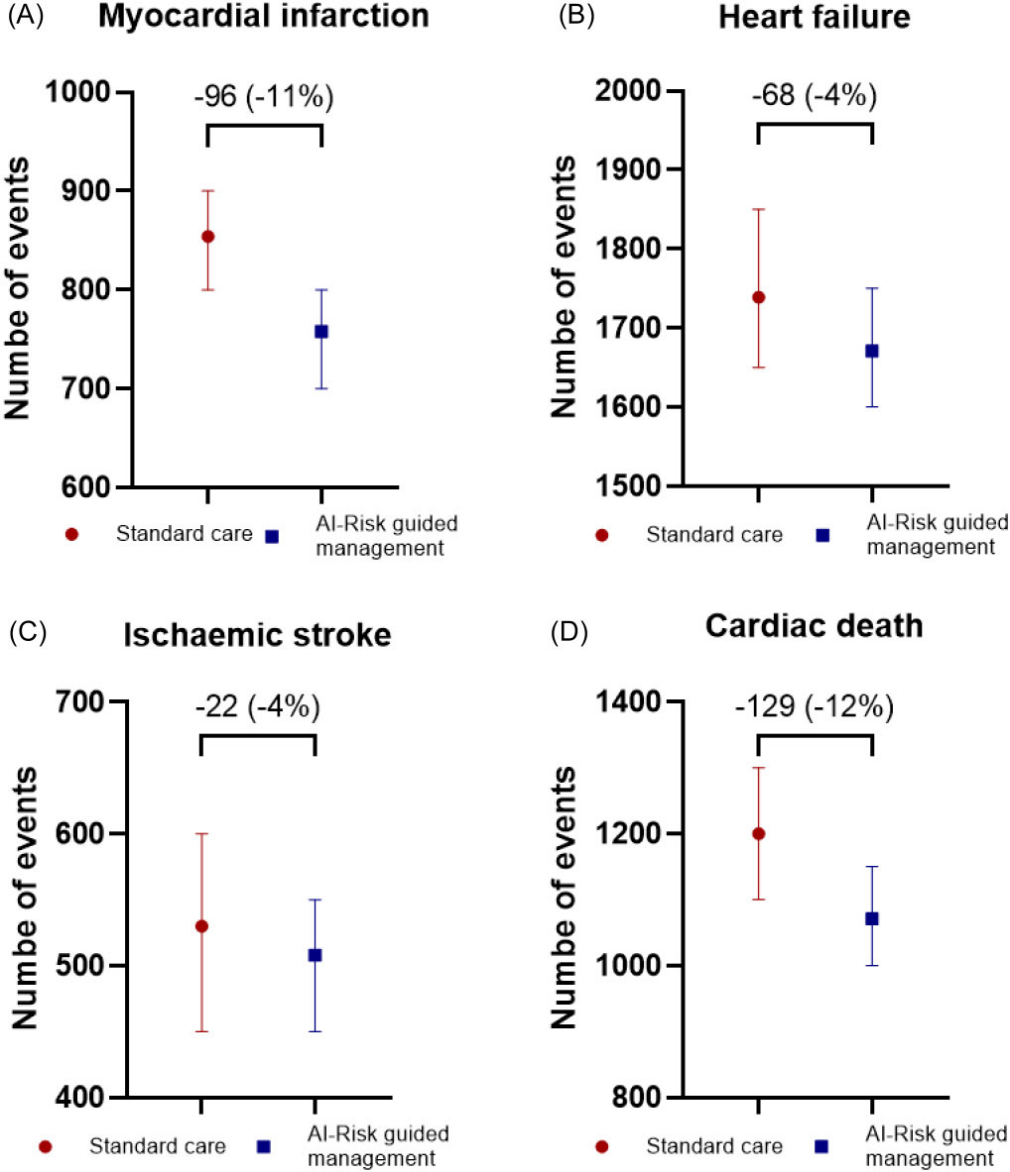
Estimated major adverse cardiac events in a cohort of 5000 simulated patients over a lifetime horizon. Data points indicate the total number of events, and bars represent 95% confidence intervals. The differences between standard care and AI-Risk guided management are expressed in absolute reduction of events (% reduction).

Implementing AI-Risk in routine clinical care (assuming full compliance) reduced the number of cardiovascular events when modelled over a lifetime horizon, with 96 fewer MI (−11%), 22 fewer strokes (−4%), 68 fewer heart failure events (−4%), and 129 fewer cardiac deaths (−12%) per 5000 simulated patients undergoing CCTA compared with standard care (*Figure 3*). These reductions in MACE led to an increase in QALYs of 0.21 (14.09 vs. 13.88). At costs of £300, £500, and £700 per AI-Risk analysis, the incremental healthcare costs were £293, £493, and £693, respectively, resulting in respective ICERs (per QALY) of £1371, £2307, and £3244 (*Table 1*). When compared to the NICE threshold for approval of technologies of £20 000–£30 000 per QALY,^19^ these results demonstrate that use of the AI-Risk model is highly cost-effective, representing value for money to the NHS.

**Table 1:** Results of the main cost–utility analysis at different price levels of AI-Risk model

	AI-Risk analysis	Standard care	Difference mean	ICER point
	mean (95% CI)	mean (95% CI)	(95% CI)	estimate (95% CI)
*AI-Risk model price: £300*				
Costs	£7563 (7029–7984)	£7270 (6698–7865)	£293 (281–304)	£1371 (1244–1569)
QALYs	14.09 (13.17–14.92)	13.88 (12.99–14.83)	0.21 (0.18–0.24)	
*AI-Risk model price: £500*				
Costs	£7762 (7271–8361)	£7270 (6698–7865)	£493 (481–504)	£2307 (2036–2596)
QALYs	14.09 (13.04–14.83)	13.88 (12.99–14.83)	0.21 (0.19–0.24)	
*AI-Risk model price: £700*				
Costs	£7962 (7380–8563)	£7270 (6698–7865)	£693 (680–705)	£3244 (2918–3627)
QALYs	14.09 (13.19–16.06)	13.88 (12.99–14.83)	0.21 (0.19–0.24)	

CI, confidence interval; ICER, incremental cost-effectiveness ratio; and QALY, quality-adjusted life year.

**Figure 4:**
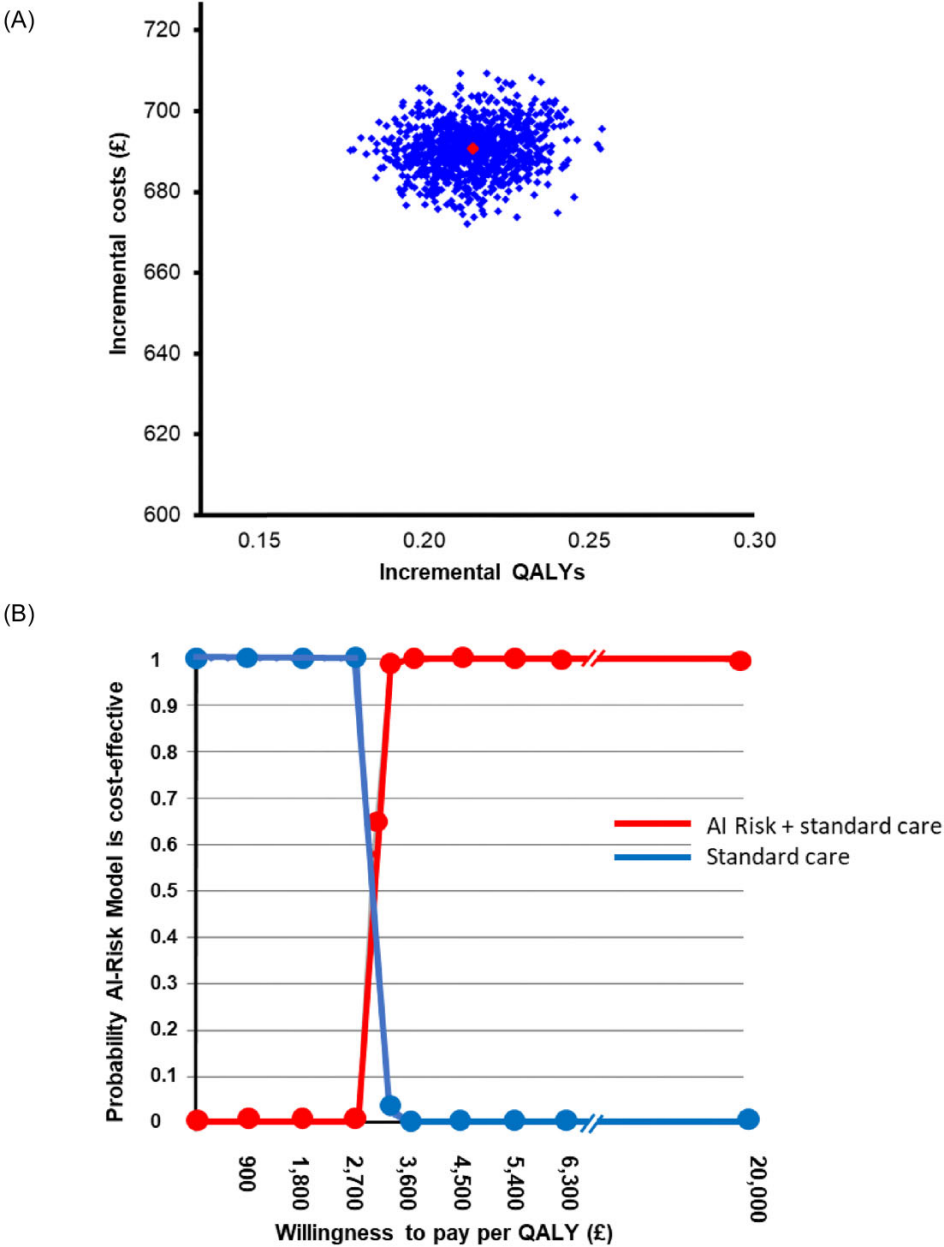
(A) Cost-effectiveness plane demonstrating the uncertainty around the incremental cost of £693 and QALY of 0.21 over 1000 sets of probabilistic sensitivity analysis, and (B) cost-effectiveness acceptability curve of AI-Risk assessment in addition to standard care compared with standard care alone assuming AI-Risk model price of £700 intercepting at an ICER of £3244. ICER, incremental cost effectiveness ratio; QALY, quality adjusted life years.

The uncertainty around the ICER at an AI-Risk model price of £700 is presented in the cost-effectiveness plane (*Figure 4A*) as well as the CEAC (*Figure 4B*) where all 1000 simulated ICERs were well below the lower NICE threshold of £20 000–£30 000/QALY.^19^ The probability of the AI-Risk model of being cost-effective was 1 at a threshold value close to £5000. The contribution of each model parameter to the uncertainty in the cost-effectiveness results is presented in the tornado plots (Supplementary material online, *Figures S3–S6*).

### Univariate scenario analyses

The results from the univariate sensitivity analyses exploring various scenarios are shown in *Table 2*, for an AI-Risk model price of £700 per analysis. When full compliance to NICE guidelines was used as comparator, the ICER was £3103/QALY gained, which was slightly lower than the ICER in the main analysis because of the more aggressive management of patients in real world clinical practice compared to the guideline-indicated management (e.g. in clinical practice patients with mild coronary atherosclerosis but no obstructive CAD often receive statin treatment while there is no such indication in the NICE guidelines). If colchicine was provided to very high-risk patients alongside statins, the estimated ICER of the AI-Risk analysis was reduced to £1837/QALY gained. Furthermore, the estimated ICER assuming implementation of the AI-Risk model only in those without obstructive CAD was £2898/QALY. When assuming the statin effect and AI-Risk analysis reclassification to be halved relative to the main analysis, the estimated ICER/QALY were £6592 and £5522, respectively. The results of all scenario analyses resulted in ICERs that were all notably lower than the traditional willingness to pay threshold of £20 000–£30 000.^19^ The budget impact analysis is presented in the Supplementary material online, *Table S7* and *Figure S7*.

**Table 2:** Results of the sensitivity analyses

	AI-Risk analysis	Standard care	Difference mean	ICER point
	mean (95% CI)	mean (95% CI)	(95% CI)	estimate (95% CI)
*AI-Risk model vs. standard care assuming full compliance with NICE guidelines*				
Costs	£7961 (7422–8582)	£7270 (6698–7865)	£691 (679–703)	£3103 (2832–3432)
QALYs	14.10 (13.22–15.10)	13.88 (12.99–14.83)	0.22 (0.20–0.25)	
*AI-Risk model vs. standard care assuming option for adding colchicine on top of satins*				
Costs	£7988 (7509–8615)	£7270 (6698–7865)	£718 (702–742)	£1837 (1662–2001)
QALYs	14.27 (13.42–15.52)	13.88 (12.99–14.83)	0.39 (0.36–0.44)	
*Model only for individuals without obstructive CAD*				
Costs	£7439 (7026–8027))	£6748 (6350–7391)	£690 (675–702)	£2898 (2658–3254)
QALYs	14.57 (13.60–15.35)	14.33 (13.37–15.10)	0.24 (0.21–0.26)	
*Model assuming 50% statin effect*				
Costs	£7988 (7290–8674)	£7270 (6698–7865)	£718 (712–722)	£6592 (5951–7270)
QALYs	13.99 (13.08–14.90)	13.88 (12.99–14.83)	0.11 (0.10–0.12)	
*Model assuming 50% of AI-Risk model on risk classification*				
Costs	£7966 (7373–8643)	£7270 (6698–7865)	£696 (691–702)	£6522 (5888–7367)
QALYs	13.99 (14.75)	13.88 (12.99–14.83)	0.11 (0.09–0.12)	

Analyses performed assuming £700 AI-model price.

CI, confidence interval; ICER, incremental cost-effectiveness ratio; and QALY, quality-adjusted life year.

## Discussion

Implementing AI-Risk Classification in clinical practice was modelled to significantly reduce cardiovascular events (MI, cardiac death, heart failure, and stroke), over a lifetime horizon. The results of the health economic analyses suggest that implementing AI-Risk Classification in addition to routine CCTA analysis for suspected CAD is cost-effective even when compared to full implementation of the NICE guidelines for cardiovascular disease risk assessment and management (NG238),^4^ with ICERs under £3500 per QALY for a wide range of prices. Importantly, AI-Risk assessment was relatively more cost-effective amongst patients without obstructive CAD on CCTA, demonstrating the value of AI-Risk to address the unmet need for tailored preventative treatment to modify the predicted risk of cardiovascular events in this patient group. Furthermore, the addition of colchicine to optimal statin treatment in the very-high risk patient group substantially improved the cost-effectiveness of AI-Risk assessment, thus facilitating the selection of patients that would benefit from further anti-inflammatory therapy from emerging clinical trial evidence such as COLCOT^21^ and LoDoCo2.^17^

The socioeconomic impact of cardiovascular disease is well established, with healthcare costs amounting to £7.4 billion per year and an annual cost of £15.8 billion to the wider economy for the UK alone.^22^ Previous economic modelling of primary prevention demonstrated that even a modest population-wide reduction in major cardiovascular events would result in a substantial cost offset and long-term health improvement (e.g. weight management programmes could yield an ICER of £2897 per QALY).^23,24^ Compared to interventions such as health promotion and pharmacotherapy, only a few studies have evaluated the cost-effectiveness of screening and risk stratification in primary prevention.^25^ In US-based studies, a strategy involving assessment of residual inflammatory risk using plasma C-reactive protein screening followed by targeted statin therapy, showed an ICER of $40 100 per QALY for 58-year old men and $87 300 per QALY for women,^26^ and statin treatment for individuals with coronary artery calcium score >0 an ICER of $18 000 per QALY in the USA.^27^ In our cost-utility analysis, AI-Risk assessment showed a lifetime ICER of £3244 per QALY compared to standard care In the UK, at an analysis cost of £700. Overall, the probability of AI-Risk assessment being cost-effective is close to 1 at a threshold value as low as £5000 per QALY, well below the lower £20 000 per QALY threshold recommended by NICE.^19^

Contemporary risk calculators use clinical risk factors to determine an individual's cardiovascular risk, and preventive measures are determined on the basis of the calculated 10-year CVD risk.^2,20^ However, these risk scores were derived from the general population and do not take into account the information from imaging tests such as CCTA, so they would likely underestimate the risk in patients with chest pain undergoing CCTA. CCTA allows direct visualization of atherosclerotic plaques, which carry the risk for acute rupture leading to MI. Whilst CCTA is used to clarify the diagnosis of CAD in these patients with chest pain, only a minority (approximately 20%) of those patients undergoing CCTA are found to have obstructive CAD.^7^ Inflammation is a key underlying pathological process that drives atherogenesis and plaque rupture, and exert deleterious effect in various types heart failure. Indeed, we have shown that quantification of coronary inflammation on CCTA helps to stratify the substantial adverse cardiac events that occur in a large number of patients without obstructive CAD.^7^ AI-Risk Classification (calculated using information derived from CCTA, capturing inflammatory risk) has demonstrated strong predictive value for (1) cardiac mortality, (2) non-fatal MI, (3) future HF, and (4) ischaemic stroke in the ORFAN study.^7^ Also, the risk for all these endpoints is significantly reduced by statin treatment in the published clinical trials.^11,14^ Indeed in the subgroup analysis of CRISP-CT study, with high FAI who did not start treatment with a statin after the test, had a HR for cardiac mortality 18.71, while patients with high FAI at baseline who started statin treatment had an HR 2.97 for cardiac mortality during the 72 months follow up.^6^ Therefore, CCTA can help us to identify the patients at risk for cardiac death, MI, HF, and stroke, and by treating these individuals with statins we can potentially reduce the incidence of these events, as described in our simulation analyses. Current clinical guidelines do not explicitly recommend initiation of statin treatment in patients after CCTA if no obstructive CAD is documented and there is no other clinical indication to do so (e.g. when QRISK3 ≥10%, familial hypercholesterolaemia, and diabetes).^20^ However, in practice many clinicians do prescribe statin treatment when mild or moderate atherosclerotic disease is documented on CCTA, even when there is no direct guideline-mandated indication. The higher statin usage seen in the real-world evaluation survey could explain the slight differences in ICERs between standard care compared with the scenario of full compliance with NICE guideline. In this study, treatment with statins at baseline as well as initiation of statin treatment based on the NICE guidelines after the conventional CCTA result, are factors taken into account, and the implementation of AI-Risk assessment adds value above and beyond standard care (which includes patients already on statins or with an indication to start statin treatment anyway), and remains a cost-effective strategy.

The strength of the AI-Risk assessment lies with its easy clinical implementation model (Software As A Service) in the standard clinical practice. The test is applicable to any routinely performed CCTA without any additional scan acquisition, hence it can be implemented even retrospectively in previously acquired CCTA scans. In practice, the CCTA digital imaging and communications in medicine file and the patient risk factors are uploaded using a secure gateway, from the picture archiving and communication system of any radiology department to a cloud-based platform (CaRi-Heart® medical device, which is a regulatory cleared for clinical use in Europe and the UK). All the analyses are performed within this environment and are reviewed/edited and cleared by appropriately qualified personnel, under the quality management system that regulates this device (EU Medical Devices Regulations-MDR) in that environment. A clinical report is then generated and returned back to the referring hospital/clinician for interpretation. Supplementary material online, *Figure S8* illustrates how the AI-Risk assessment integrates into routine clinical care, and the changes it triggers in patient management are presented in Supplementary material online, *Figure S2*.

### Limitations

Although all the scenarios consistently showed that AI-Risk assessment was a cost-effective strategy, there are several important assumptions and limitations. First, the current study used effect estimates from a prospective cohort in the ORFAN study rather than a randomized controlled trial. However, as an offset to any disadvantages from absence of randomization, the cohort study captures consecutive patients undergoing clinically indicated CCTA, who are more representative of a real-world clinical population. Linkage of outcomes data with national registries also allowed minimal loss to follow-up, thus providing an accurate lifetime projection. Second, the costs were evaluated using UK figures and practices. The AI-Risk algorithm was initially trained on a US patient cohort and validated in a European population.^10^ It was further externally validated in a large cohort of the UK population.^7^ Given the comparable clinical indications for CCTA and uniformity in subsequent management across multiple international guidelines,^3,20,28^ we would expect cost-effectiveness to be consistent in multiple jurisdictions. However, further studies might be warranted to evaluate the cost-effectiveness of AI-Risk assessment in different geographical areas. Third, the models in this study assumed that AI-Risk assessment was performed in all CCTAs. AI-Risk assessment could be retrospectively applied to CCTAs that have already been performed in routine clinical practice without further image acquisition. It is therefore feasible to fully implement the AI-Risk assessment in routine CCTA reporting to provide risk stratification. Fourth, in the health economic analysis, only changes in treatment with statin and colchicine were considered. Moreover, prospective changes of medication over the lifetime of the individuals have not been considered. Finally, the risk of subsequent MACE was assumed to be the same as for the first event. In practice, the occurrence of a MACE increases the risk of future events. As such, the protective effects of improved management due to the AI-Risk model are likely to be underestimated.

## Conclusions

In this study we have demonstrated the incremental health and cost benefit of AI-Risk assessment, by modelling the observed clinical outcomes over a lifetime horizon and therapeutic effect of changes in medical therapy. AI-Risk assessment reclassifies patients and changes management by improving the accuracy of CVD risk prediction. The deployment of this image analysis method in clinical practice would refine risk assessment, guiding additional preventive cardiovascular treatments, while it is likely to be cost-effective compared with standard care.

## Supplementary Material

qcae085_Supplemental_File

## Data Availability

Unit cost data used in this analysis are available in the article and online supplementary material.
