# Supplementary material for: Cost-effectiveness of a novel AI technology to quantify coronary inflammation and cardiovascular risk in patients undergoing routine coronary computed tomography angiography
Source: Eur Heart J Qual Care Clin Outcomes. 2024 Sep 28;11(4):434–44. doi: 10.1093/ehjqcco/qcae085 (PMC12187117; doi:10.1093/ehjqcco/qcae085)
Supplement: qcae085_Supplemental_File [file qcae085_supplemental_file.docx]

**Supplementary material**

**Cost-effectiveness of a novel AI technology to quantify coronary inflammation and cardiovascular risk in patients undergoing routine Coronary Computed Tomography Angiography**

Apostolos Tsiachristas^1*^, Kenneth Chan^2*^, Elizabeth Wahome^2*^, Ben Kearns^3^, Parijat Patel^2^, Maria Lyasheva^2^, Nigar Syed^4^, Sam Fry^4^, Thomas Halborg^2^, Henry West^2,5^, Ed Nicol^6,7^, David Adlam^8,9^, Bhavik Modi^8,9^, Attila Kardos^10^, John P. Greenwood^11,12^, Nikant Sabharwal^2^, Giovanni Luigi De Maria^2^, Shahzad Munir^13^, Elisa McAlindon^13^, Yogesh Sohan^4^, Pete Tomlins^4^, Muhammad Siddique^4^, Cheerag Shirodaria^2,4^, Ron Blankstein^14^, Milind Desai^15^, Stefan Neubauer^2^, Keith M. Channon^2^, John Deanfield^16^, Ron Akehurst^3^, Charalambos Antoniades^2^ on behalf of the ORFAN Consortium

1. Nuffield Department of Primary Care Health Sciences & Department of Psychiatry, University of Oxford, Oxford, United Kingdom

2. Acute Multidisciplinary Imaging & Interventional Centre, British Heart Foundation (BHF) Centre of Research Excellence, Division of Cardiovascular Medicine, Radcliffe Department of Medicine, Oxford NIHR Biomedical Research Centre, University of Oxford, United Kingdom

3. Lumanity, Sheffield, United Kingdom

4. Caristo Diagnostics, Oxford, United Kingdom

5. Sydney Medical Schol, University of Sydney, Sydney, Australia

6. Departments of Cardiology and Radiology, Royal Brompton Hospital, London, United Kingdom

7. School of Biomedical Engineering and Imaging Sciences, King’s College, London, London, United Kingdom

8. Department of Cardiovascular Sciences, University of Leicester and NIHR Leicester Biomedical Research Centre, Leicester, United Kingdom

9. NIHR Leicester Biomedical Research Centre, Leicester, United Kingdom

10. Department of Cardiology, Translational Cardiovascular Research Group, Milton Keynes University Hospital NHS Foundation Trust, Milton Keynes, United Kingdom

11. Leeds Teaching Hospitals, Leeds, United Kingdom

12. Baker Heart and Diabetes Institute, Melbourne, Australia

13. Royal Wolverhampton NHS Trust, Wolverhampton, United Kingdom

14. Brigham and Women's Hospital, Harvard Medical School, Boston, MA, USA

15. Department of Cardiovascular Medicine, Cleveland Clinic Heart Vascular and Thoracic Institute, Cleveland, OH, USA

16. Institute of Cardiovascular Science, University College London, United Kingdom

*authors equally contributed to the study

**Correspondence to: Professor** Charalambos Antoniades MD PhD FRCP FESC,

Acute Multidisciplinary Imaging & Interventional Centre (AMIIC), University of Oxford, John Radcliffe Hospital, Oxford OX3 9DU.

e-mail: [charalambos.antoniades@cardiov.ox.ac.uk](mailto:charalambos.antoniades@cardiov.ox.ac.uk)

**Index of supplementary materials**

**Description of the observational studies used ………………………..………………………… Page 3**

**Healthcare cost and utility measures……………………………………………..……………… Page 5**

**Table S1 ICD-10 and OPCS-4 terms used to define clinical states.....………… …..………..… Page 6**

**Figure S1. Study design of prospective evaluation of AI-Risk assessment against full
compliance to current guidelines and real-world evaluation of AI-risk assessment………… Page 7**

**Figure S2. Overview of the proposed clinical care pathway based on the AI-Risk
Classification categories derived from interpretation of FAI Score and AI-Risk…………… Page 8**

**Figure S3. Baseline characteristics of patient cohorts………………………………………… Page 9**

**Table S3. Full list of model input parameters of the economic model……………………… Page 10-16**

**Table S4. Summary of statin effect on outcomes by risk group and treatment
initialization/intensification …………………………………………………………………… Page 17**

**Table S5. Full calculation of statin effect…………………………………………………… Page 18**

**Table S6. Cost inflation calculation………………………………………………………… Page 19**

# Figure S3: Tornado diagram with parameters of statin effect on incremental cost effectiveness ratio (ICER). ……………………………………… Page 20

# Supplementary Figure S4: Tornado diagram with parameters of transition probability on incremental cost effectiveness ratio (ICER) ……………………………………………. Page 21

**Supplementary Figure S5: Tornado diagram with parameters of risk classification on incremental cost effectiveness ratio (ICER). ………………………………………………………………………. Page 22**

**Supplementary Figure S6: Tornado diagram with parameters of cost and utility on incremental cost effectiveness ratio (ICER). ………………………………………………………………………. Page 23**

**Supplementary figure S7. Budget impact analysis for deployment of the AI-Risk classification in the UK National Health System. ………………………………………………………………………… Page 24**

**Figure S8: Overview of the software as a service (SAAS) system for delivery of
FAI-Score and AI-Risk in clinical practice. …………………………………………………… Page 25**

**Supplementary table S7. Budget Impact Calculations for deployment of the
AI-Risk classification ……………………………………………………………………..……. Page 26-27**

**Supplementary references……………………………………………………………………… Page 28**

**CHEERS 2022 Checklist for the health economic evaluation ………………………………. Page 29-30**

**Description of the patient cohorts included**

***Long-term outcomes cohort study***

The Oxford Risk Factors and Non-invasive Imaging Study (ORFAN) study (Clinical Trials.gov: NCT05169333) is a multicentre cohort study of patients who has undergone routine clinical CCTA. Nested cohorts within the United Kingdom were used in the current study. CCTA images were transferred to the Acute Multidisciplinary Imaging and Interventional Centre (AMIIC) core lab at the University of Oxford for analyses. Baseline clinical data and clinical outcome events were collected from local hospital electronic patient record, as well as through linkage with national registries including NHS Digital SSNAP (Sentinel Stroke National Audit Program) and NICOR (National Institute for Cardiovascular Outcomes Research). ICD-10 codes were used to extract clinical diagnosis such as myocardial infarction, ischaemic stroke, heart failure and cardiac mortality (Supplementary table S1). Procedures were defined based on Office of Population Censuses and Surveys’ Classification of Surgical Operations version 4 (OPCS-4) codes. (Supplementary table S1) All events in the ORFAN study were adjudicated with cross-validation between NHS England dataset, NICOR and SSNAP. The ORFAN study was approved by the Oxfordshire Research Ethics Committee (REC 15/SC/0545). Patient information was collected under Section 251 of the National Health Service Act 2006, following approval by the Confidentiality Advisory Group (20/CAG/0157). All CCTA images were transferred to the Oxford core lab for analysis. CCTAs indicated for investigation of congenital heart disease (n=91), cardiac transplant (n=103), or poor image quality (n=74) were excluded (Figure S1)^1^.

***Prospective real-world evaluation survey of AI-Risk-guided management vs current clinical practice***

To evaluate the impact of AI-Risk assessment on clinical management, a prospective, real-world evaluation study was performed. Data were made available from four National Health Service (NHS) Hospitals that were considered representative of the population of UK (Oxford University Hospitals NHS Foundation Trust, Milton Keynes University Hospital NHS Foundation Trust, University Hospitals of Leicester NHS Foundation Trust and Royal Wolverhampton NHS Trust). Analyses were performed prospectively using the CaRi-Heart V2.5 device in 744 consecutive patients undergoing CCTA as part of clinical care for the diagnosis of coronary artery disease (CAD) during 2021-2022.

A total of 9 consultant cardiologists who are independent to the study team were involved in the evaluation of clinical management. The list of information made available to the clinical care teams included patient demographics (Age, sex, ethnicity), cardiovascular risk factors (diabetes, smoking status, hypertension, hypercholesterolaemia), baseline medications, baseline past medical histories, clinical risk scores (QRISK3, QRISK3 category, European Society of cardiology SCORE, ESC SCORE category), conventional CCTA interpretations (clinical report, extend of coronary atherosclerosis and CADRADS2.0 categories).

Treatment recommendations as per standard clinical care were then recorded, including lifestyle advice, initiation/intensification of medical treatments (statin, aspirin, colchicine or any other medications, and treatment details (name and dose).

The AI-Risk assessment report was then presented to the same clinician, including fat-attenuation index score, absolute AI-Risk and AI-Risk classification. Treatment recommendations were then recorded again, including lifestyle advice, adding or altering medical therapy (statin, aspirin, colchicine or any other medications, and treatment details (name and dose)), changes in follow-up plans, and whether there was improved confidence in the treatment decisions. The outcomes of the real-world evaluation were analysed by the local clinical care teams who provided aggregated results to the research team.

***Prospective clinical study*** ***of AI-Risk-guided management vs*** ***fully implemented clinical guidelines.***

Since clinical management may vary depending on local practices, a further prospective study was performed to allow state-of-the-art risk assessment of patients undergoing CCTA, assuming full compliance of the clinical care team with the NICE guidelines.^5-7^ Consecutive patients undergoing CCTA as part of clinical care (n=1,214) were prospectively recruited in three NHS hospitals (Oxford University Hospitals, University Hospitals of Leicester, Milton Keynes Hospital) during 2016-2022 as part of the prospective study arm of ORFAN study. Blood samples were collected at the time of CCTA, and plasma lipids were measured to facilitate accurate, centralised calculation of QRISK3 for the primary analysis. Demographics, clinical risk factors, and renal function were documented. The CCTA scans were analysed using the CaRi-Heart v2.5 device and the risk classification based on the FAI Score and AI-Risk results was compared to QRISK3. The QRISK3 classification is recommended by the current NICE guidelines and estimates the 10-year risk of a cardiovascular event (fatal or non-fatal), classifying patients as low/medium risk if <10%, high risk if 10 to <20% or very high risk if ≥20%.^8^ Patients at high- or very-high risk have an indication to start pharmacological treatments for risk factor management, often leading to initiation or dose intensification of statin treatment. QRISK3 was calculated using age, sex, ethnicity, smoking, diabetes, lipid profile, family history, chronic kidney disease, atrial fibrillation, blood pressure measurement, migraine, rheumatoid arthritis, systemic lupus erythematosus, severe mental illness, antipsychotic medication, steroid tablets, body mass index and lipid profile [https://qrisk.org/].^9^ The reclassification and recording of potential change in management were performed by the research team, against the state-of-the-art implementation of NICE guidance for primary and secondary prevention.^5,6^ Informed consent was obtained from all participants, and the study was approved by the Oxfordshire Research Ethics Committee (ORFAN study 15/SC/0545).

**Statistical power calculations**

In the prospective long-term outcome cohort, Cox-proportional model was used for survival analysis. To detect hazard ratio of 1.2 with α set at 0.05 & power of 90%, 1276 patients are required. Therefore, the observed outcomes from the median 7.7 year follow up in the prospective outcomes cohort (n=3,393) would provide sufficient power for simulation of clinical outcome over a lifetime horizon. Power calculations in health economic evaluations was addressed early on in the development of cost-effectiveness analysis (Briggs and Gray 1998)^10^. In cost-effectiveness analysis, the joint distribution of cost and effects is the subject of analysis rather than testing a hypothesis of detecting a pre-defined difference in a primary outcome. Uncertainty in treatment effects as well as all other parameters in the model were explored by performing probability sensitivity analysis (i.e. drawing values of the distributions of each model parameter for thousands of iterations and estimating incremental costs and incremental QALY in each iteration). In our model, we have performed probabilistic sensitivity analysis using 5,000 iterations, and based on these, we provided 95%CIs for the estimated incremental costs and incremental QALYs following good practice in conducting cost-effectiveness analysis. This analysis provided us with high certainty that the intervention is cost-effective i.e. none of the 5,000 ICERs resulted from the probabilistic sensitivity analysis was above the £20,000 threshold that NICE recommends to determine cost-effectiveness.

**Healthcare cost and utility measures**

The costs in each health state were derived from three UK studies and included resource use in primary and secondary care.^11-13^ All costs were inflated from their base year to 2022 values using the Hospital and Community Health Service (HCHS) pay and price inflation. The cost of statin treatment (i.e. 40mg and 80mg of atorvastatin) was derived from the British National Formulary and applied to a patients’ lifetime, assuming full adherence once it had been initiated (**Supplementary table** **S2**). The detailed inflation calculations are presented in **Supplementary table S5**. For each health state, a utility score was assigned based on the most recent UK based literature.^14^ The utility scores assigned to each health state are presented in **Supplementary table S2**. For cardiac mortality and other death health states the utility score was set to 0. Quality-adjusted life years (QALYs) were calculated by applying the area-under-the-curve method.

The time that cardiologists spent in training to interpret AI-Risk analyses and delivering it to patients was recorded and monetised using the unit cost of a cardiologist’s workday. The average intervention training costs to the NHS of adding AI-Risk assessment to CCTA per patient were calculated by dividing the total intervention training costs by the total number of patients with an AI-Risk assessment result. However, from the real-world evaluation study the time that cardiologists spent on AI-Risk assessment training was 1 hour on average. Assuming that each cardiologist would interpret around 320 CCTAs per year (i.e. 8 patients per week for 40 weeks per year) the intervention costs (i.e. in addition to the AI-Risk assessment costs) are negligible and therefore were excluded from the analysis. Using the results of the main analysis at a £300, £500 and £700 price per AI-based risk scan and assuming 200,000 CCTA scans yearly in the UK, we estimated the net impact that the implementation of AI-Risk model would have on the NHS budget. The budget impact was estimated over 5 years and the annual uptake of AI-Risk model in the NHS was assumed to be 2%, 5%, 10%, 15%, and 20% in that period.

## **Budget Impact analysis**

The impact of the AI-Risk Model (at £700 price) on the NHS budget over 5 years was estimated to be £2,821,971 respectively in the first year of its introduction to the NHS (assuming 2% national uptake) followed by a budget impact of £7,076,651 (assuming 5% uptake), £14,182,648 (assuming 10% uptake), £21,992,618 (assuming 15% uptake), and £30,169,955 (assuming 20% uptake), in the next consecutive years. This is illustrated in **Supplementary figure 4** and the detailed calculations are presented in **Supplementary table S6**.

**Supplementary table S1:** ICD-10 and OPCS-4 terms used to define clinical states.

|  |  | **ICD-10 Codes** | **OPCS-4 Codes** |
| --- | --- | --- | --- |
| **Events** | |  |  |
|  | Myocardial infarction | STEMI [I21.1, I21.2, I21.3, I22.0, I22.1, I22.8], NSTEMI [I21.4, I21.9, I22.9] and unspecified MI [I21.0, I23.X, I24.1, I24.8, I24.9] |  |
|  | Heart failure | I11.0, I13.0, I13.2, I25.5, I42.0, I42.5, I42.8, I42.9, I50.0, I50.1, I50.9 K76.1 |  |
|  | Stroke | I63 |  |
|  | Cardiac mortality | I11, I13, I20, I21, I22, I23, I24, I25, I30, I31, I32, I33, I34, I35, I36, I37, I38, I39, I40, I41, I42, I43, I44, I45, I46, I47, I48, I49, I50, I51, I52 |  |
| **Procedures** | |  |  |
|  | PCI |  | K49.x-50.x, K75.x |
|  | CABG |  | K4[0123456] [123489], K45[56], K4[6]5 |
|  |  |  |  |
| **Risk factors** | |  |  |
|  | Hypertension | I10.x, I11.x, I12.x, I13.x, I15.x |  |
|  | Hyperlipidaemia | E78.0, E78.1, E78.2, E78.4, E78.5 |  |
|  | Diabetes mellitus | E11.x |  |
|  | Atrial fibrillation | I48.x |  |
|  | Stroke | I63.x, I65.x, I66.x |  |
|  | Valvular heart disease | I05.x, I06.x, I07.x, I08.0, I08.1, I08.2, I08.3, I34.x, I35.x, I36.x, I37.x, I39.0, I39.1, I39.3 |  |

*STEMI= ST-elevation myocardial infarction, NSTEMI= Non-ST-elevation myocardial infarction, PCI=Percutaneous coronary intervention, CABG= Coronary artery bypass graft*


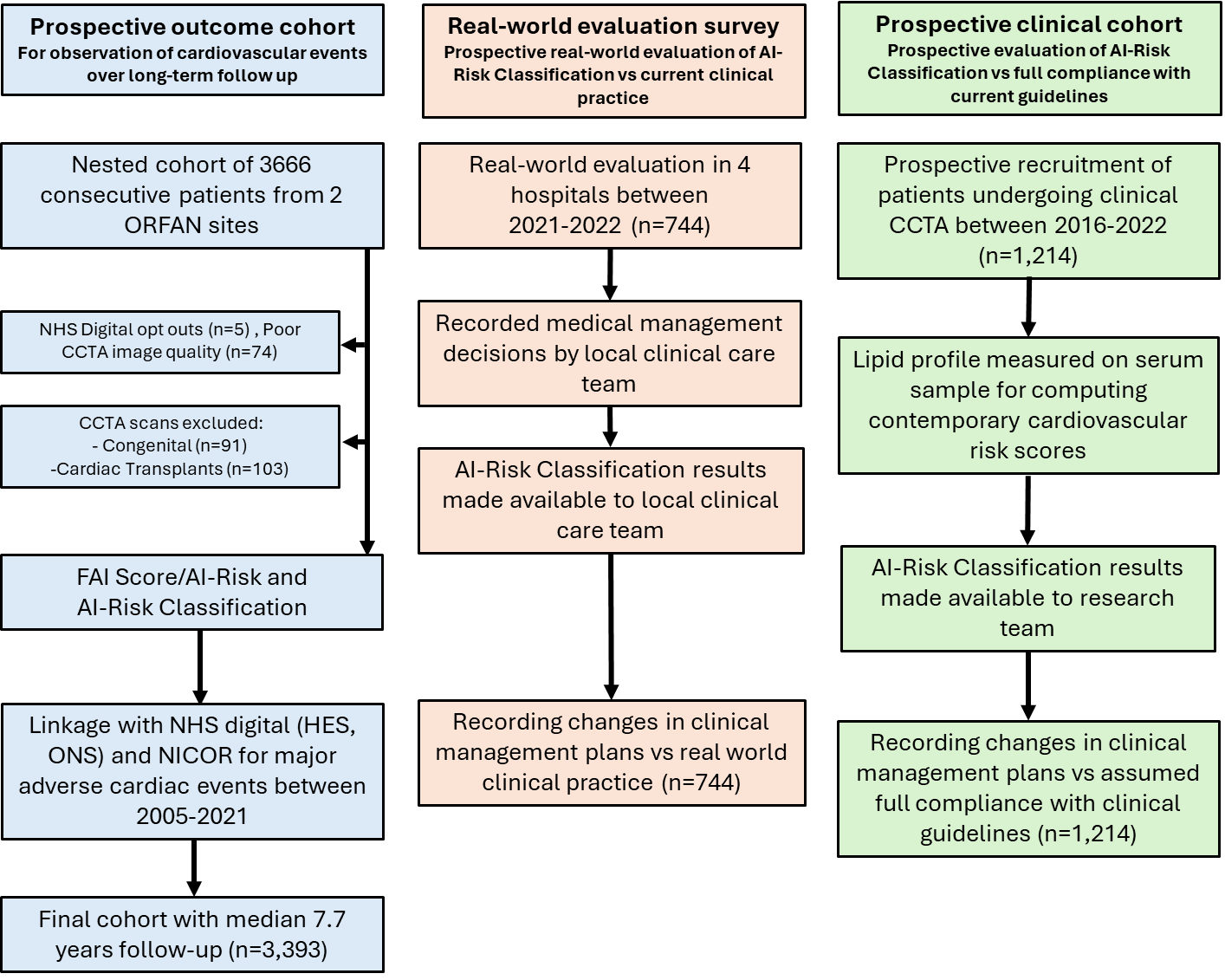


**Supplementary figure S1.** Study design of prospective evaluation of AI-Risk assessment against full compliance to current guidelines and real-world evaluation of AI-risk assessment


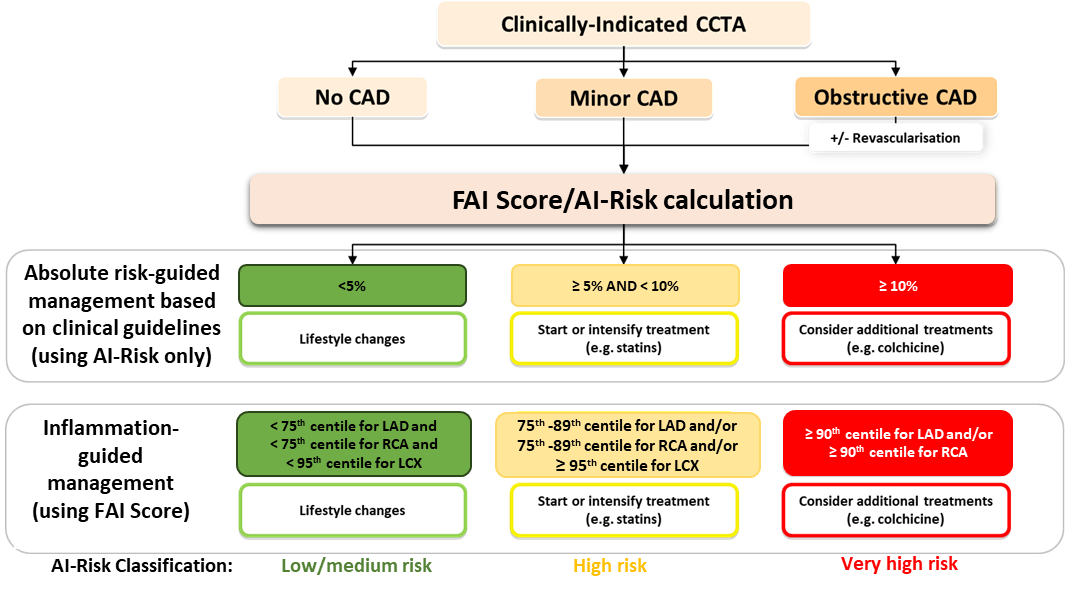


**Supplementary figure S2.** Overview of the proposed clinical care pathway based on the AI-Risk Classification categories derived from interpretation of FAI Score and AI-Risk. Modified from Oikonomou EO et al Cardiovasc Res 2021 (117), 2677–2690. FAI score analysis is performed in the LAD, RCA and LCX. Analysis of the perivascular fat around the left main stem is avoided because of the high anatomical variability in that part of the coronary tree.

**Supplementary table S2.** Baseline characteristics of study cohorts

| **Characteristics** | | **Prospective clinical study**  **(n = 1,214)** | | **Real-world evaluation survey**  **(n = 744)** | |
| --- | --- | --- | --- | --- | --- |
|  |  | **n (%)** | | **n (%)** | |
| **Demographics** | |  |  |  |  |
| Age, years (IQR) | | 59 | (52-66) | 75 | (65-82) |
| Male | | 672 | (55.4) | 514 | (69.3) |
| Ethnicity | |  |  |  |  |
|  | White | 1,132 | (93.2) | 475 | (63.8) |
|  | Asian or Asian British | 64 | (5.3) | 64 | (8.6) |
|  | Black or Black British | 10 | (0.8) | 8 | (1.1) |
|  | Other groups | 6 | (0.5) | 6 | (0.8) |
|  | Unknown | 2 | (0.2) | 191 | (25.7) |
| **Cardiovascular risk factors** | |  |  |  |  |
| Smoking | | 124 | (10.2) | 94 | (12.6) |
| Hypertension | | 507 | (41.8) | 295 | (39.7) |
| Hypercholesterolemia | | 345 | (28.4) | 272 | (36.6) |
| Diabetes mellitus | | 98 | (8.1) | 110 | (14.8) |
| **Obstructive CAD on CCTA** | | 107 | (8.8) | 107 | (14.4) |
| **QRISK3 score category** | |  |  |  |  |
|  | Low/medium risk (<10%) | 804 | (66.2) | 400 | (53.8) |
|  | High risk (10-19%) | 359 | (29.6) | 215 | (28.9) |
|  | Very high risk (≥20%) | 51 | (4.2) | 129 | (17.3) |
| **AI-Risk Classification** | |  |  |  |  |
|  | Low/medium risk | 696 | (57.3) | 183 | (24.6) |
|  | High risk | 254 | (20.9) | 162 | (21.8) |
|  | Very high risk | 264 | (21.7) | 399 | (53.6) |
| **Medications** | |  |  |  |  |
| Antiplatelets | | 286 | (23.6) | 183 | (24.6) |
| Beta blockers | | 295 | (24.3) | 180 | (24.2) |
| Calcium channel blockers | | 201 | (16.6) | 115 | (15.5) |
| Nitrates | | 126 | (10.4) | 24 | (3.2) |
| Statins | | 371 | (30.6) | 303 | (40.7) |
| ACE inhibitors | | 223 | (18.4) | 114 | (15.3) |
| Angiotensin receptor blockers | | 100 | (8.2) | 61 | (8.2) |
| Diuretics | | 95 | (7.8) | 89 | (12.0) |
| Digoxin | | 5 | (0.4) | 3 | (0.4) |
| Insulin | | 18 | (1.5) | 20 | (2.7) |
| Oral hypoglycaemics | | 63 | (5.2) | 70 | (9.4) |

CAD, coronary artery disease; CCTA, coronary computed tomography angiogram;

IQR, Interquartile range

**Supplementary table S3.** Full list of model input parameters of the economic model

| **Description** | **Value** | **SEM** | **distribution** | **Source** |
| --- | --- | --- | --- | --- |
| Costs |  |  |  |  |
| AI-risk model price | £300/  £500/  £700 |  | Gamma | Assumption |
| Cost of 40mg statin | 4 | 0.0 | Gamma | BNF |
| Cost in CAD health state | 641 | 32.1 | Gamma | Danese et al., 2016 (Table 2)^11^ |
| Cost of cardiac death | 1500 | 75.0 | Gamma | Zhou et al., 2023 (Table 4)^13^ |
| Cost in heart failure health state | 2261 | 113.1 | Gamma | Danese et al., 2016 (Table 2)^11^ |
| Cost in MI health state | 3003 | 150.2 | Gamma | Danese et al., 2016 (Table 2)^11^ |
| Cost of non-cardiac death | 2503 | 125.2 | Gamma | Keng et al., 2022 (Figure 2)^12^ |
| Cost in stroke health state | 2488 | 124.4 | Gamma | Danese et al., 2016 (Table 2)^11^ |
| **Utilities** |  |  |  |  |
| Utility in CAD health state | 0.750 | 0.038 | Beta | Blieden Betts et al., 2020 (Table 2, EQ-5D, stable angina)^14^ |
| Utility in heart failure health state | 0.680 | 0.034 | Beta | Kraai et al 2013 (Table 2, EQ-5D, total group)^15^ |
| Utility in MI health state | 0.718 | 0.036 | Beta | Sterne et al., 2017 (Table 14)^16^ |
| Utility in no CAD health state | 0.779 | 0.039 | Beta | Sterne et al., 2017 (Table 14)^16^ |
| Utility in stroke health state | 0.650 | 0.033 | Beta | Blieden Betts et al., 2020 (Table 2, EQ-5D, post-stroke)^14^ |
| **Risk level** |  |  |  |  |
| Proportion of having CAD | 0.11 | 0.012 | Beta | Real-world evaluation survey |
| Proportion of people with CAD at very high risk | 0.68 | 0.057 | Beta | Real-world evaluation survey |
| Proportion of people with CAD at high risk | 0.25 | 0.053 | Beta | Real-world evaluation survey |
| Proportion of people without CAD at very high risk | 0.48 | 0.095 | Beta | Real-world evaluation survey |
| Proportion of people without CAD at high risk | 0.22 | 0.045 | Beta | Real-world evaluation survey |
| **AI-Risk model effect** |  |  |  | Real-world evaluation survey |
| Proportion of people with CAD at very high risk initiating statin due to AI-risk model | 0.02 | 0.001 | Beta | Real-world evaluation survey |
| Proportion of people with CAD at very high risk intensifying statin dose due to AI-risk model | 0.19 | 0.001 | Beta | Real-world evaluation survey |
| Proportion of people with CAD at low/medium risk initiating statin due to AI-risk model | 0.00 | 0.000 | Beta | Real-world evaluation survey |
| Proportion of people with CAD at low/medium risk intensifying statin dose due to AI-risk model | 0.00 | 0.000 | Beta | Real-world evaluation survey |
| Proportion of people with CAD at high risk initiating statin due to AI-risk model | 0.00 | 0.000 | Beta | Real-world evaluation survey |
| Proportion of people with CAD at high risk intensifying statin dose due to AI-risk model | 0.07 | 0.004 | Beta | Real-world evaluation survey |
| Proportion of people without CAD at very high risk initiating statin due to AI-risk model | 0.34 | 0.017 | Beta | Real-world evaluation survey |
| Proportion of people without CAD at very high risk intensifying statin dose due to AI-risk model | 0.22 | 0.001 | Beta | Real-world evaluation survey |
| Proportion of people without CAD at very low/medium risk initiating statin due to AI-risk model | 0.06 | 0.003 | Beta | Real-world evaluation survey |
| Proportion of people without CAD at low/medium risk intensifying statin dose due to AI-risk model | 0.03 | 0.002 | Beta | Real-world evaluation survey |
| Proportion of people without CAD at high risk initiating statin due to AI-risk model | 0.42 | 0.021 | Beta | Real-world evaluation survey |
| Proportion of people without CAD at high risk intensifying statin dose due to AI-risk model | 0.16 | 0.008 | Beta | Real-world evaluation survey |
| **Statin effect size based on level of risk** |  |  |  |  |
| Relative Risk of cardiac death of initiating statin in people with CAD | 0.80 | 0.004 | Log-Normal | CTT Lancet 2005^17^ & 2012^18^ |
| Relative Risk of cardiac death of intensifying statin in people with CAD | 0.98 | 0.005 | Log-Normal | CTT Lancet 2005^17^ & 2012^18^ |
| Relative Risk of cardiac death of initiating statin in people without CAD | 0.75 | 0.004 | Log-Normal | CTT Lancet 2005^17^ & 2012^18^ |
| Relative Risk of cardiac death of intensifying statin in people without CAD | 0.97 | 0.005 | Log-Normal | CTT Lancet 2005^17^ & 2012^18^ |
| Relative Risk of MI of initiating statin in people at very high risk | 0.57 | 0.003 | Log-Normal | CTT Lancet 2005^17^ & 2012^18^ |
| Relative Risk of MI of intensifying statin in people at very high risk | 0.94 | 0.005 | Log-Normal | CTT Lancet 2005^17^ & 2012^18^ |
| Relative Risk of MI of initiating statin in people at low/medium risk | 0.25 | 0.001 | Log-Normal | CTT Lancet 2005^17^ & 2012^18^ |
| Relative Risk of MI of intensifying statin in people at low/medium risk | 0.87 | 0.004 | Log-Normal | CTT Lancet 2005^17^ & 2012^18^ |
| Relative Risk of MI of initiating statin in people at high risk | 0.40 | 0.002 | Log-Normal | CTT Lancet 2005^17^ & 2012^18^ |
| Relative Risk of MI of intensifying statin in people at high risk | 0.91 | 0.005 | Log-Normal | CTT Lancet 2005^17^ & 2012^18^ |
| Relative Risk of non-cardiac death of initiating statin in people with CAD | 0.93 | 0.005 | Log-Normal | CTT Lancet 2005^17^ & 2012^18^ |
| Relative Risk of non-cardiac death of intensifying statin in people with CAD | 0.99 | 0.005 | Log-Normal | CTT Lancet 2005^17^ & 2012^18^ |
| Relative Risk of non-cardiac death of initiating statin in people without CAD | 0.75 | 0.004 | Log-Normal | CTT Lancet 2005^17^ & 2012^18^ |
| Relative Risk of non-cardiac death of intensifying statin in people without CAD | 0.97 | 0.005 | Log-Normal | CTT Lancet 2005^17^ & 2012^18^ |
| Relative Risk of stroke of initiating statin in people at high risk | 0.77 | 0.004 | Log-Normal | CTT Lancet 2005^17^ & 2012^18^ |
| Relative Risk of stroke of intensifying statin in people at very high risk | 0.97 | 0.005 | Log-Normal | CTT Lancet 2005^17^ & 2012^18^ |
| Relative Risk of stroke of initiating statin in people at low/medium risk | 0.59 | 0.003 | Log-Normal | CTT Lancet 2005^17^ & 2012^18^ |
| Relative Risk of stroke of intensifying statin in people at low/medium risk | 0.95 | 0.005 | Log-Normal | CTT Lancet 2005^17^ & 2012^18^ |
| Relative Risk of stroke of initiating statin in people at high risk | 0.63 | 0.003 | Log-Normal | CTT Lancet 2005^17^ & 2012^18^ |
| Relative Risk of stroke of intensifying statin in people at high risk | 0.95 | 0.005 | Log-Normal | CTT Lancet 2005^17^ & 2012^18^ |
| **Transition probabilities** |  |  |  |  |
| Probability of cardiac death in the CAD health state in people at very high risk | 0.017 | 0.000 | Beta | Long-term outcomes cohort study^3^ |
| Probability of heart failure in the CAD health state in people at very high risk | 0.027 | 0.000 | Beta | Long-term outcomes cohort study^3^ |
| Probability of MI in the CAD health state in people at very high risk | 0.016 | 0.000 | Beta | Long-term outcomes cohort study^3^ |
| Probability of non-cardiac death in the CAD health state in people at very high risk | 0.019 | 0.000 | Beta | Long-term outcomes cohort study^3^ |
| Probability of stroke in the CAD health state in people at very high risk | 0.004 | 0.000 | Beta | Long-term outcomes cohort study^3^ |
| Probability of cardiac death in the CAD health state in people at low/medium risk | 0.004 | 0.000 | Beta | Long-term outcomes cohort study^3^ |
| Probability of heart failure in the CAD health state in people at low/medium risk | 0.007 | 0.000 | Beta | Long-term outcomes cohort study^3^ |
| Probability of MI in the CAD health state in people at low/medium risk | 0.009 | 0.000 | Beta | Long-term outcomes cohort study^3^ |
| Probability of non-cardiac death in the CAD health state in people at low/medium risk | 0.009 | 0.000 | Beta | Long-term outcomes cohort study^3^ |
| Probability of stroke in the CAD health state in people at low/medium risk | 0.002 | 0.000 | Beta | Long-term outcomes cohort study^3^ |
| Probability of non-cardiac death in the CAD health state in people at high risk | 0.007 | 0.000 | Beta | Long-term outcomes cohort study^3^ |
| Probability of heart failure in the CAD health state in people at high risk | 0.012 | 0.000 | Beta | Long-term outcomes cohort study^3^ |
| Probability of MI in the CAD health state in people at high risk | 0.015 | 0.000 | Beta | Long-term outcomes cohort study^3^ |
| Probability of non-cardiac death in the CAD health state in people at high risk | 0.014 | 0.000 | Beta | Long-term outcomes cohort study^3^ |
| Probability of stroke in the CAD health state in people at high risk | 0.002 | 0.000 | Beta | Long-term outcomes cohort study^3^ |
| Probability of not having a MACE in the CAD health state in people at very high risk | 0.034 | 0.000 | Beta | Long-term outcomes cohort study^3^ |
| Probability of not having a MACE in the CAD health state in people at low/medium risk | 0.028 | 0.000 | Beta | Long-term outcomes cohort study^3^ |
| Probability of not having a MACE in the CAD health state in people at high risk | 0.030 | 0.000 | Beta | Long-term outcomes cohort study^3^ |
| Probability of cardiac death in the no-CAD health state in people at very high risk | 0.005 | 0.000 | Beta | Long-term outcomes cohort study^3^ |
| Probability of heart failure in the no-CAD health state in people at very high risk | 0.011 | 0.000 | Beta | Long-term outcomes cohort study^3^ |
| Probability of MI in the no-CAD health state in people at very high risk | 0.003 | 0.000 | Beta | Long-term outcomes cohort study^3^ |
| Probability of non-cardiac death in the no-CAD health state in people at very high risk | 0.012 | 0.000 | Beta | Long-term outcomes cohort study^3^ |
| Probability of stroke in the no-CAD health state in people at very high risk | 0.003 | 0.000 | Beta | Long-term outcomes cohort study^3^ |
| Probability of cardiac death in the no-CAD health state in people at low/medium risk | 0.001 | 0.000 | Beta | Long-term outcomes cohort study^3^ |
| Probability of heart failure in the no-CAD health state in people at low/medium risk | 0.003 | 0.000 | Beta | Long-term outcomes cohort study^3^ |
| Probability of MI in the no-CAD health state in people at low/medium high risk | 0.002 | 0.000 | Beta | Long-term outcomes cohort study^3^ |
| Probability of non-cardiac death in the no-CAD health state in people at low/medium risk | 0.003 | 0.000 | Beta | Long-term outcomes cohort study^3^ |
| Probability of stroke in the no-CAD health state in people at low/medium risk | 0.001 | 0.000 | Beta | Long-term outcomes cohort study^3^ |
| Probability of cardiac death in the no-CAD health state in people at high risk | 0.002 | 0.000 | Beta | Long-term outcomes cohort study^3^ |
| Probability of heart failure in the no-CAD health state in people at high risk | 0.007 | 0.000 | Beta | Long-term outcomes cohort study^3^ |
| Probability of MI in the no-CAD health state in people at high risk | 0.003 | 0.000 | Beta | Long-term outcomes cohort study^3^ |
| Probability of non-cardiac death in the no-CAD health state in people at high risk | 0.006 | 0.000 | Beta | Long-term outcomes cohort study^3^ |
| Probability of stroke in the no-CAD health state in people at high risk | 0.001 | 0.000 | Beta | Long-term outcomes cohort study^3^ |
| **Sensitivity analysis: NICE guidelines** |  |  |  |  |
| Proportion of people with CAD at very high risk initiating statin due to AI-risk model | 0.13 | 0.007 | Beta | Prospective clinical study |
| Proportion of people with CAD at very high risk intensifying statin dose due to AI-risk model | 0.13 | 0.007 | Beta | Prospective clinical study |
| Proportion of people with CAD at low/medium risk initiating statin due to AI-risk model | 0.00 | 0.000 | Beta | Prospective clinical study |
| Proportion of people with CAD at low/medium risk intensifying statin dose due to AI-risk model | 0.00 | 0.000 | Beta | Prospective clinical study |
| Proportion of people with CAD at high risk initiating statin due to AI-risk model | 0.33 | 0.017 | Beta | Prospective clinical study |
| Proportion of people with CAD at high risk intensifying statin dose due to AI-risk model | 0.00 | 0.000 | Beta | Prospective clinical study |
| Proportion of people without CAD at very high risk initiating statin due to AI-risk model | 0.37 | 0.019 | Beta | Prospective clinical study |
| Proportion of people without CAD at very high risk intensifying statin dose due to AI-risk model | 0.20 | 0.010 | Beta | Prospective clinical study |
| Proportion of people without CAD at very low/medium risk initiating statin due to AI-risk model | 0.00 | 0.000 | Beta | Prospective clinical study |
| Proportion of people without CAD at low/medium risk intensifying statin dose due to AI-risk model | 0.00 | 0.000 | Beta | Prospective clinical study |
| Proportion of people without CAD at high risk initiating statin due to AI-risk model | 0.45 | 0.022 | Beta | Prospective clinical study |
| Proportion of people without CAD at high risk intensifying statin dose due to AI-risk model | 0.23 | 0.011 | Beta | Prospective clinical study |
| **Sensitivity analysis: Colchicine** |  |  |  |  |
| Cost of colchicine | 2.84 | 0.142 | Gamma | BNF |
| For those at high risk, the colchicine effect was multiplied with the risk of MACEs after having statin i.e. an additional risk protection from colchicine to MACEs | 0.80 | 0.04 | Beta | Assumption 20% additional relative risk reduction |
| **Sensitivity analysis: Reduced statin effect by 50%** |  |  |  |  |
| Relative Risk of cardiac death of initiating statin in people with CAD | 0.90 | 0.004 | Log-Normal | Assumption: 50% reduction in effect reported in CTT Lancet 2005^17^ & 2012^18^ |
| Relative Risk of cardiac death of intensifying statin in people with CAD | 0.99 | 0.005 | Log-Normal | Assumption: 50% reduction in effect reported in CTT Lancet 2005^17^ & 2012^18^ |
| Relative Risk of cardiac death of initiating statin in people without CAD | 0.87 | 0.004 | Log-Normal | Assumption: 50% reduction in effect reported in CTT Lancet 2005^17^ & 2012^18^ |
| Relative Risk of cardiac death of intensifying statin in people without CAD | 0.99 | 0.005 | Log-Normal | Assumption: 50% reduction in effect reported in CTT Lancet 2005^17^ & 2012^18^ |
| Relative Risk of MI of initiating statin in people at very high risk | 0.79 | 0.003 | Log-Normal | Assumption: 50% reduction in effect reported in CTT Lancet 2005^17^ & 2012^18^ |
| Relative Risk of MI of intensifying statin in people at very high risk | 0.97 | 0.005 | Log-Normal | Assumption: 50% reduction in effect reported in CTT Lancet 2005^17^ & 2012^18^ |
| Relative Risk of MI of initiating statin in people at low/medium risk | 0.63 | 0.001 | Log-Normal | Assumption: 50% reduction in effect reported in CTT Lancet 2005^17^ & 2012^18^ |
| Relative Risk of MI of intensifying statin in people at low/medium risk | 0.93 | 0.004 | Log-Normal | Assumption: 50% reduction in effect reported in CTT Lancet 2005^17^ & 2012^18^ |
| Relative Risk of MI of initiating statin in people at high risk | 0.70 | 0.002 | Log-Normal | Assumption: 50% reduction in effect reported in CTT Lancet 2005^17^ & 2012^18^ |
| Relative Risk of MI of intensifying statin in people at high risk | 0.95 | 0.005 | Log-Normal | Assumption: 50% reduction in effect reported in CTT Lancet 2005^17^ & 2012^18^ |
| Relative Risk of non-cardiac death of initiating statin in people with CAD | 0.97 | 0.005 | Log-Normal | Assumption: 50% reduction in effect reported in CTT Lancet 2005^17^ & 2012^18^ |
| Relative Risk of non-cardiac death of intensifying statin in people with CAD | 1.00 | 0.005 | Log-Normal | Assumption: 50% reduction in effect reported in CTT Lancet 2005^17^ & 2012^18^ |
| Relative Risk of non-cardiac death of initiating statin in people without CAD | 0.87 | 0.004 | Log-Normal | Assumption: 50% reduction in effect reported in CTT Lancet 2005^17^ & 2012^18^ |
| Relative Risk of non-cardiac death of intensifying statin in people without CAD | 0.99 | 0.005 | Log-Normal | Assumption: 50% reduction in effect reported in CTT Lancet 2005^17^ & 2012^18^ |
| Relative Risk of stroke of initiating statin in people at high risk | 0.88 | 0.004 | Log-Normal | Assumption: 50% reduction in effect reported in CTT Lancet 2005^17^ & 2012^18^ |
| Relative Risk of stroke of intensifying statin in people at very high risk | 0.99 | 0.005 | Log-Normal | Assumption: 50% reduction in effect reported in CTT Lancet 2005^17^ & 2012^18^ |
| Relative Risk of stroke of initiating statin in people at low/medium risk | 0.79 | 0.003 | Log-Normal | Assumption: 50% reduction in effect reported in CTT Lancet 2005^17^ & 2012^18^ |
| Relative Risk of stroke of intensifying statin in people at low/medium risk | 0.97 | 0.005 | Log-Normal | Assumption: 50% reduction in effect reported in CTT Lancet 2005^17^ & 2012^18^ |
| Relative Risk of stroke of initiating statin in people at high risk | 0.81 | 0.003 | Log-Normal | Assumption: 50% reduction in effect reported in CTT Lancet 2005^17^ & 2012^18^ |
| Relative Risk of stroke of intensifying statin in people at high risk | 0.98 | 0.005 | Log-Normal | Assumption: 50% reduction in effect reported in CTT Lancet 2005^17^ & 2012^18^ |
| **Sensitivity analysis: Reduced AI-Risk model effect by 50%** |  |  |  |  |
| Proportion of people with CAD at very high risk initiating statin due to AI-risk model | 0.01 | 0.001 | Beta | Assumption: 50% reduction in effect reported in real-world evaluation survey |
| Proportion of people with CAD at very high risk intensifying statin dose due to AI-risk model | 0.09 | 0.005 | Beta | Assumption: 50% reduction in effect reported in real-world evaluation survey |
| Proportion of people with CAD at low/medium risk initiating statin due to AI-risk model | 0.00 | 0.000 | Beta | Assumption: 50% reduction in effect reported in real-world evaluation survey |
| Proportion of people with CAD at low/medium risk intensifying statin dose due to AI-risk model | 0.00 | 0.000 | Beta | Assumption: 50% reduction in effect reported in real-world evaluation survey |
| Proportion of people with CAD at high risk initiating statin due to AI-risk model | 0.00 | 0.000 | Beta | Assumption: 50% reduction in effect reported in real-world evaluation survey |
| Proportion of people with CAD at high risk intensifying statin dose due to AI-risk model | 0.04 | 0.002 | Beta | Assumption: 50% reduction in effect reported in real-world evaluation survey |
| Proportion of people without CAD at very high risk initiating statin due to AI-risk model | 0.17 | 0.009 | Beta | Assumption: 50% reduction in effect reported in real-world evaluation survey |
| Proportion of people without CAD at very high risk intensifying statin dose due to AI-risk model | 0.11 | 0.005 | Beta | Assumption: 50% reduction in effect reported in real-world evaluation survey |
| Proportion of people without CAD at very low/medium risk initiating statin due to AI-risk model | 0.03 | 0.002 | Beta | Assumption: 50% reduction in effect reported in real-world evaluation survey |
| Proportion of people without CAD at low/medium risk intensifying statin dose due to AI-risk model | 0.02 | 0.001 | Beta | Assumption: 50% reduction in effect reported in real-world evaluation survey |
| Proportion of people without CAD at high risk initiating statin due to AI-risk model | 0.21 | 0.011 | Beta | Assumption: 50% reduction in effect reported in real-world evaluation survey |
| Proportion of people without CAD at high risk intensifying statin dose due to AI-risk model | 0.08 | 0.004 | Beta | Assumption: 50% reduction in effect reported in real-world evaluation survey |

BNF, British National Formulary; CAD, Coronary artery disease; CTT, Cholesterol Treatment Trialists; HF, Heart failure; MACE, Major adverse cardiac events; MI, Myocardial infarction; SEM, standard error of mean

**Supplementary table S4.** Summary of statin effect on outcomes by risk group and treatment initialization/intensification

| **Statin effect on outcomes** | **Very high risk** | **High risk** | **Low / medium risk** | **Very high risk** | **High risk** | **Low / medium risk** |
| --- | --- | --- | --- | --- | --- | --- |
|  | **Initiate** | | | **Intensify** | | |
| MI | 57% | 40% | 25% | 94% | 91% | 87% |
| Stroke | 77% | 63% | 59% | 97% | 95% | 95% |
| Cardiac death; patients with CAD | 80% | | | 98% | | |
| Cardiac death; patients with no CAD | 75% | | | 97% | | |
| Non-cardiac death; patients with CAD | 93% | | | 99% | | |
| Non-cardiac death; patients with no CAD | 75% | | | 97% | | |

CAD, coronary artery disease; MI, Myocardial infarction

| **Changes per unit of LDL reduction** | | | | | | | | **Moderate intensity (Atorvastatin 40)** | | | | | **Intensify from 40mg to 80 mg** | | | | |
| --- | --- | --- | --- | --- | --- | --- | --- | --- | --- | --- | --- | --- | --- | --- | --- | --- | --- |
|  | RR per 1mmol/L LDL-C reduction | 95% CI low | 95% CI high | Ln(RR per 1 mmol) | ln(LB) | ln(HB) | ln(se) per 1mmol/L LDL C reduction | Ln(RR) | ln(se) | **RR for use in model** | **95% CI low** | **95% CI high** | Ln(RR) | ln(se) | **RR for use in model** | **95% CI low** | **95% CI high** |
| **DEATHS** |  |  |  |  |  |  |  |  |  |  |  |  |  |  |  |  |  |
| *No obstructive CAD* |  |  |  |  |  |  |  |  |  |  |  |  |  |  |  |  |  |
| Vascular death in <5% risk | 0.80 | 0.43 | 1.47 | -0.22 | -0.84 | 0.39 | 0.31 | -0.40 | 0.56 | **0.67** | **0.23** | **2.00** | -0.04 | 0.06 | **0.96** | **0.86** | **1.08** |
| Vascular death in 5-10% risk | 0.75 | 0.55 | 1.04 | -0.29 | -0.60 | 0.04 | 0.16 | -0.51 | 0.29 | **0.60** | **0.34** | **1.06** | -0.05 | 0.03 | **0.95** | **0.89** | **1.01** |
| Vascular death in 10-20% risk | 0.84 | 0.67 | 1.05 | -0.17 | -0.40 | 0.05 | 0.11 | -0.31 | 0.20 | **0.73** | **0.49** | **1.09** | -0.03 | 0.02 | **0.97** | **0.93** | **1.01** |
| Vascular death all risks | 0.85 | 0.77 | 0.95 | -0.16 | -0.26 | -0.05 | 0.05 | -0.29 | 0.10 | **0.75** | **0.62** | **0.90** | -0.03 | 0.01 | **0.97** | **0.95** | **0.99** |
| Non-Vascular death in <5% risk | 1.13 | 0.76 | 1.69 | 0.12 | -0.27 | 0.52 | 0.20 | 0.22 | 0.36 | **1.24** | **0.61** | **2.53** | 0.02 | 0.04 | **1.02** | **0.95** | **1.10** |
| Non-Vascular death in 5-10% risk | 0.87 | 0.67 | 1.11 | -0.14 | -0.40 | 0.10 | 0.13 | -0.25 | 0.23 | **0.78** | **0.50** | **1.22** | -0.03 | 0.02 | **0.97** | **0.93** | **1.02** |
| Non-Vascular death in 10-20% risk | 0.94 | 0.76 | 1.15 | -0.06 | -0.27 | 0.14 | 0.11 | -0.11 | 0.19 | **0.90** | **0.62** | **1.29** | -0.01 | 0.02 | **0.99** | **0.95** | **1.03** |
| Non-Vascular death all risks | 0.97 | 0.88 | 1.07 | -0.16 | -0.26 | -0.05 | 0.05 | -0.29 | 0.10 | **0.75** | **0.62** | **0.90** | -0.03 | 0.01 | **0.97** | **0.95** | **0.99** |
| *CAD* |  |  |  |  |  |  |  |  |  |  |  |  |  |  |  |  |  |
| Vascular death in <5% risk | 0.93 | 0.53 | 1.62 | -0.07 | -0.63 | 0.48 | 0.29 | -0.13 | 0.51 | **0.88** | **0.33** | **2.37** | -0.01 | 0.05 | **0.99** | **0.89** | **1.09** |
| Vascular death in 5-10% risk | 1.07 | 0.81 | 1.41 | 0.07 | -0.21 | 0.34 | 0.14 | 0.12 | 0.25 | **1.13** | **0.69** | **1.84** | 0.01 | 0.03 | **1.01** | **0.96** | **1.07** |
| Vascular death in 10-20% risk | 0.89 | 0.79 | 1 | -0.12 | -0.24 | - | 0.06 | -0.21 | 0.11 | **0.81** | **0.66** | **1.00** | -0.02 | 0.01 | **0.98** | **0.96** | **1.00** |
| Vascular death all risks | 0.88 | 0.84 | 0.92 | -0.13 | -0.17 | -0.08 | 0.02 | -0.23 | 0.04 | **0.80** | **0.74** | **0.86** | -0.02 | 0.00 | **0.98** | **0.97** | **0.98** |
| Non-Vascular death in <5% risk | 1.38 | 0.53 | 3.63 | 0.32 | -0.63 | 1.29 | 0.49 | 0.57 | 0.87 | **1.77** | **0.32** | **9.78** | 0.06 | 0.09 | **1.06** | **0.89** | **1.27** |
| Non-Vascular death in 5-10% risk | 0.92 | 0.61 | 1.41 | -0.08 | -0.49 | 0.34 | 0.21 | -0.15 | 0.38 | **0.86** | **0.41** | **1.81** | -0.02 | 0.04 | **0.98** | **0.91** | **1.06** |
| Non-Vascular death in 10-20% risk | 0.95 | 0.81 | 1.11 | -0.05 | -0.21 | 0.10 | 0.08 | -0.09 | 0.14 | **0.91** | **0.69** | **1.21** | -0.01 | 0.01 | **0.99** | **0.96** | **1.02** |
| Non-Vascular death all risks | 0.96 | 0.9 | 1.02 | -0.04 | -0.11 | 0.02 | 0.03 | -0.07 | 0.06 | **0.93** | **0.83** | **1.04** | -0.01 | 0.01 | **0.99** | **0.98** | **1.00** |
| **Other MACEs** |  |  |  |  |  |  |  |  |  |  |  |  |  |  |  |  |  |
| Nonfatal MI in <5% risk | 0.46 | 0.28 | 0.76 | -0.78 | -1.27 | -0.27 | 0.25 | -1.38 | 0.45 | **0.25** | **0.10** | **0.61** | -0.14 | 0.05 | **0.87** | **0.79** | **0.95** |
| Nonfatal MI in 5-10% risk | 0.6 | 0.49 | 0.74 | -0.51 | -0.71 | -0.30 | 0.11 | -0.91 | 0.19 | **0.40** | **0.28** | **0.58** | -0.09 | 0.02 | **0.91** | **0.88** | **0.95** |
| Nonfatal MI in >10% risk | 0.73 | 0.65 | 0.83 | -0.31 | -0.43 | -0.19 | 0.06 | -0.56 | 0.11 | **0.57** | **0.46** | **0.71** | -0.06 | 0.01 | **0.94** | **0.92** | **0.97** |
| Stroke in <5% | 0.74 | 0.46 | 1.19 | -0.30 | -0.78 | 0.17 | 0.24 | -0.53 | 0.43 | **0.59** | **0.25** | **1.36** | -0.06 | 0.04 | **0.95** | **0.87** | **1.03** |
| Stroke in 5-10% | 0.77 | 0.6 | 0.98 | -0.26 | -0.51 | -0.02 | 0.13 | -0.46 | 0.22 | **0.63** | **0.41** | **0.97** | -0.05 | 0.02 | **0.95** | **0.91** | **1.00** |
| Stroke in >10% | 0.86 | 0.75 | 0.98 | -0.15 | -0.29 | -0.02 | 0.07 | -0.27 | 0.12 | **0.77** | **0.60** | **0.97** | -0.03 | 0.01 | **0.97** | **0.95** | **1.00** |

**Supplementary table S5:** Full calculations for statin effect

CI, confidence intervals; CTT LB, lower boundary; MACE, major adverse cardiac event; RR relative risk reduction; UB, upper boundary

**Supplementary table S6.** Cost inflation calculation

|  | Original cost | Cost base year | Source | Inflation factor (2022) | Inflated cost | Converted to cycle length |
| --- | --- | --- | --- | --- | --- | --- |
| Obstructive CAD | £2,263 | 2014 | Danese et al., 2016 (Table 2)^11^ | 1.134 | £2,566 | £641 |
| Cardiac death | £1,460 | 2020 | Zhou et al., 2023 (Table 4)^13^ | 1.027 | £1,500 | NA |
| HF | £3,989 | 2014 | Danese et al., 2016 (Table 2)^11^ | 1.134 | £4,523 | £2,261 |
| MI | £5,298 | 2014 | Danese et al., 2016 (Table 2)^11^ | 1.134 | £6,006 | £3,003 |
| No obstructive CAD | 0 |  |  |  |  |  |
| Non-cardiac death | £2437 | 2020 | Keng et al., 2022 (Figure 2)^12^ | 1.0272 | £2,503 | NA |
| Stroke | £4,389 | 2014 | Danese et al., 2016 (Table 2)^11^ | 1.134 | £4,977 | £2,488 |

CAD: coronary artery disease; HF: heart failure; MI: Myocardial infarction

**
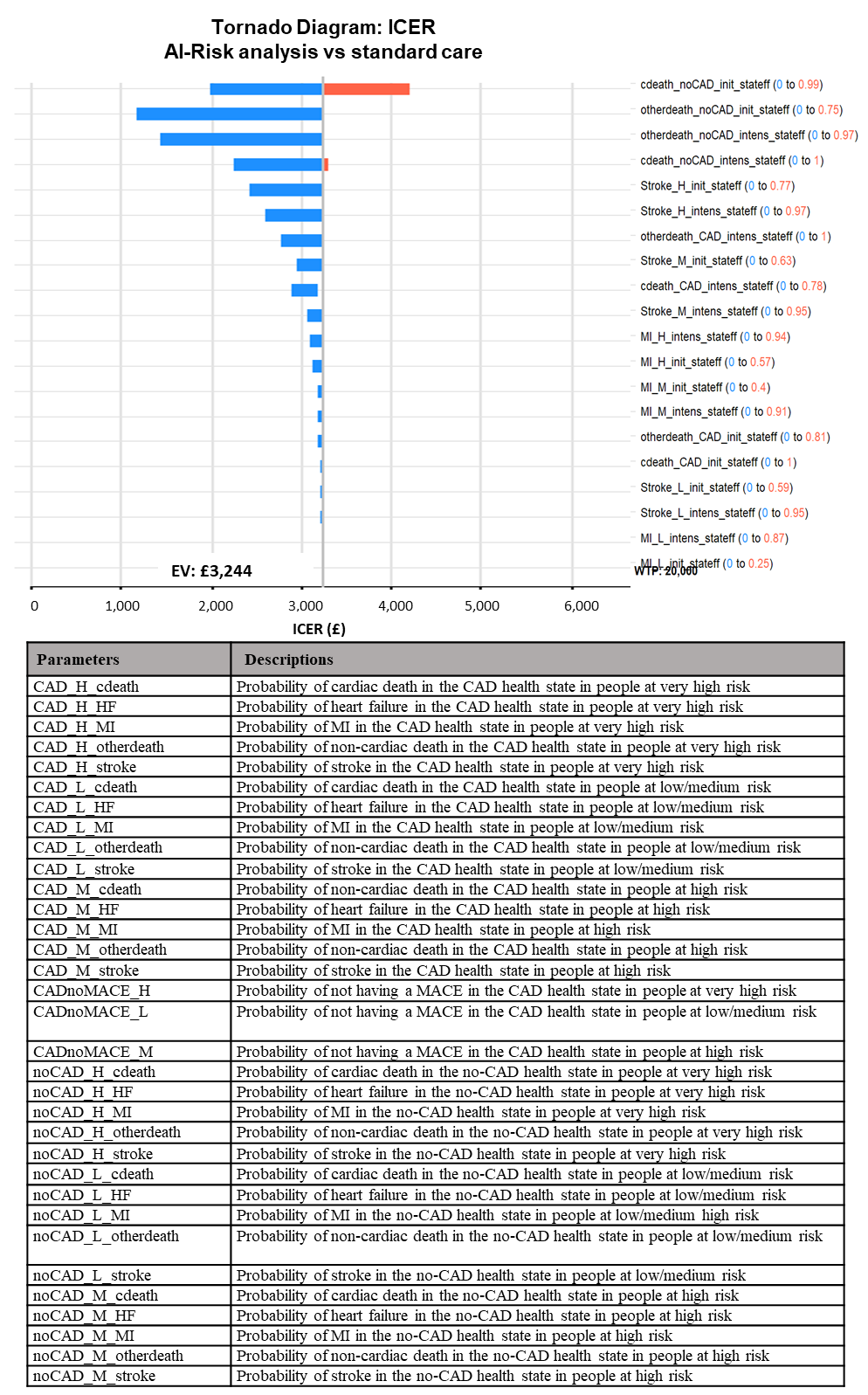
**

**Supplementary Figure S3:** Tornado diagram with parameters of statin effect on incremental cost effectiveness ratio (ICER). After removing each model parameter, the uncertainty around the expected value (EV) of £3,244 were all well under the willingness to pay (WTP) threshold of £20,000.

**
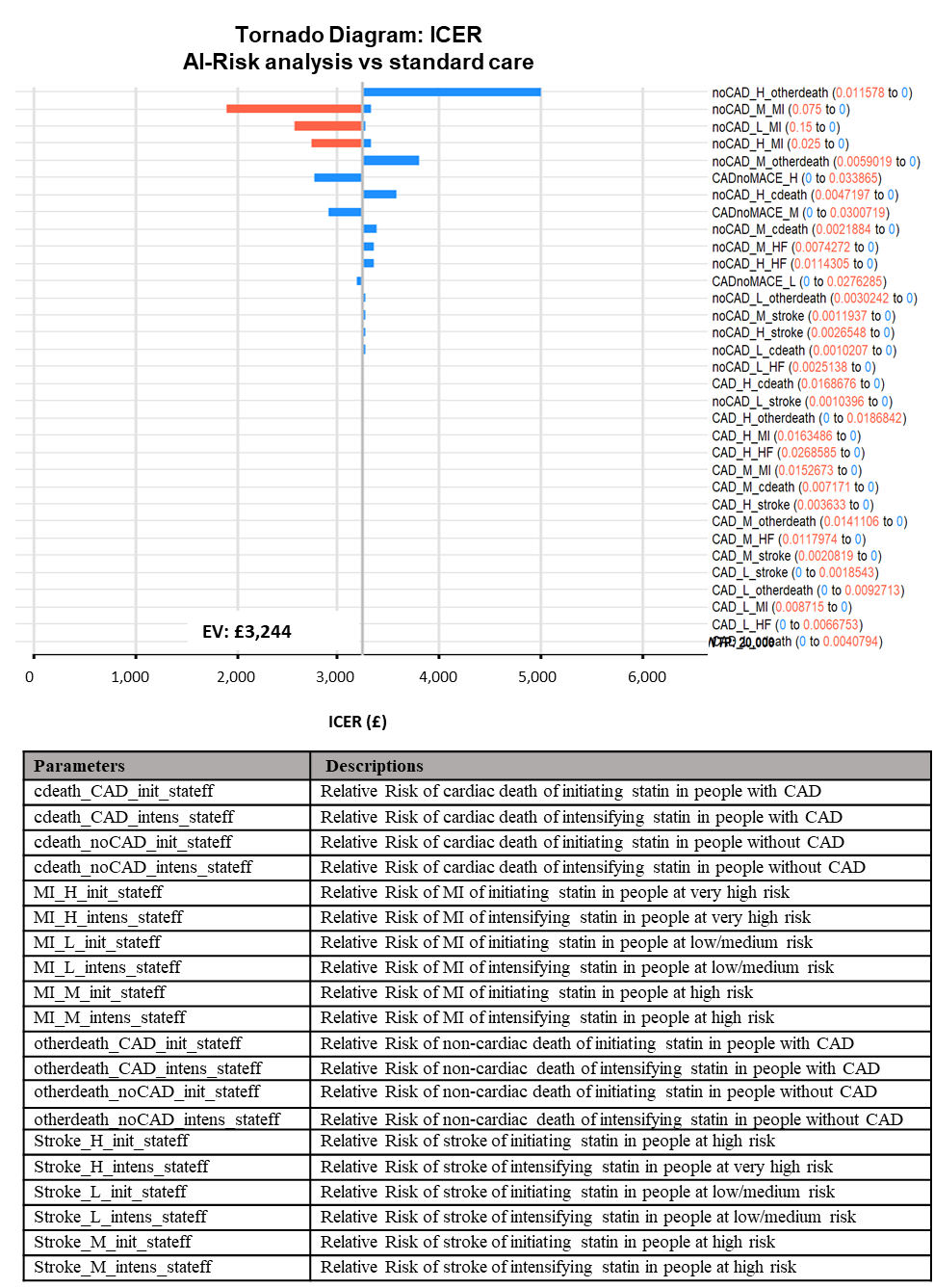
**

**Supplementary Figure S4:** Tornado diagram with parameters of transition probability on incremental cost effectiveness ratio (ICER). After removing each model parameter, the uncertainty around the expected value (EV) of £3,244 were all well under the willingness to pay (WTP) threshold of £20,000.

**
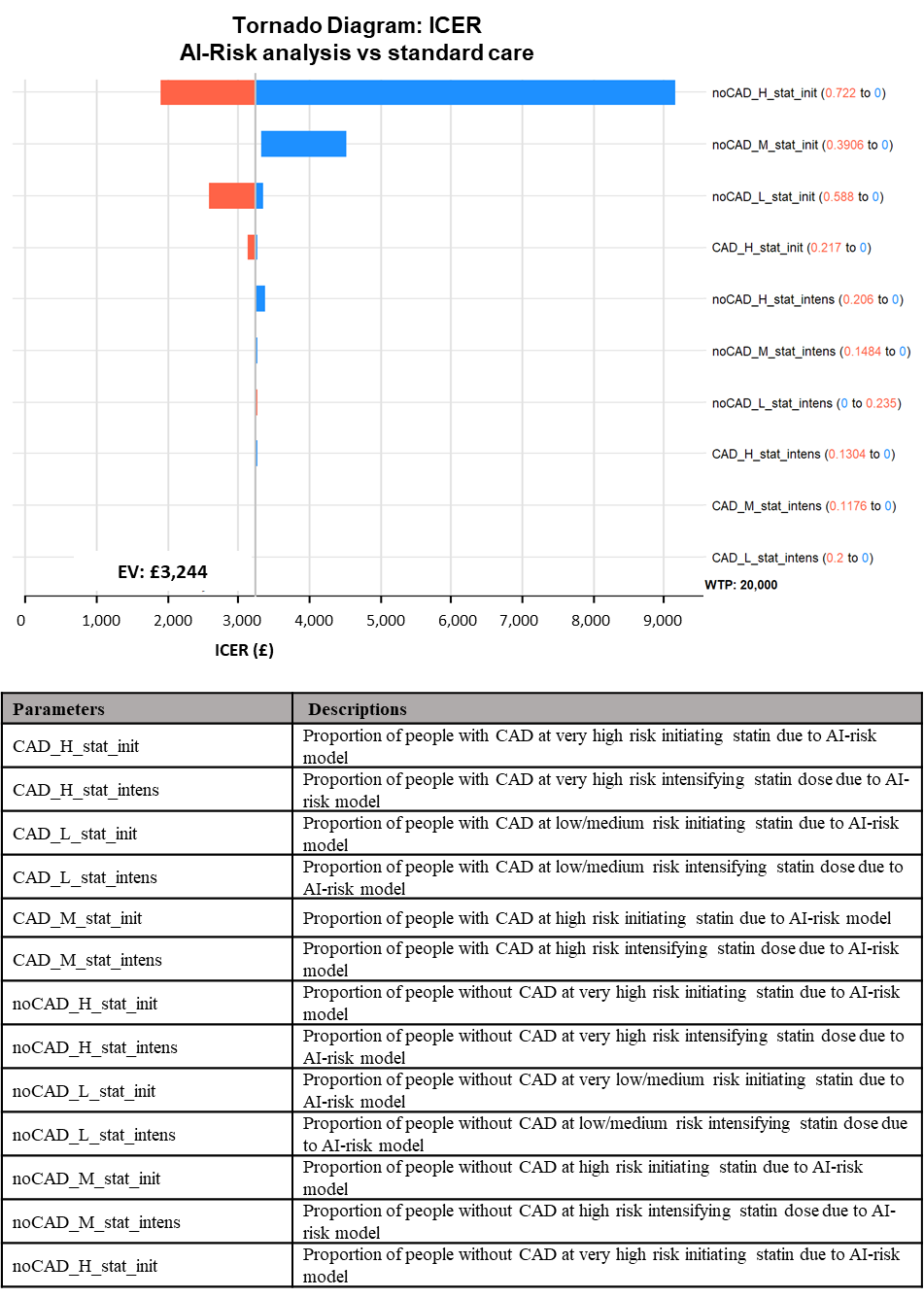
**

**Supplementary Figure S5:** Tornado diagram with parameters of risk classification on incremental cost effectiveness ratio (ICER). After removing each model parameter, the uncertainty around the expected value (EV) of £3,244 were all well under the willingness to pay (WTP) threshold of £20,000.


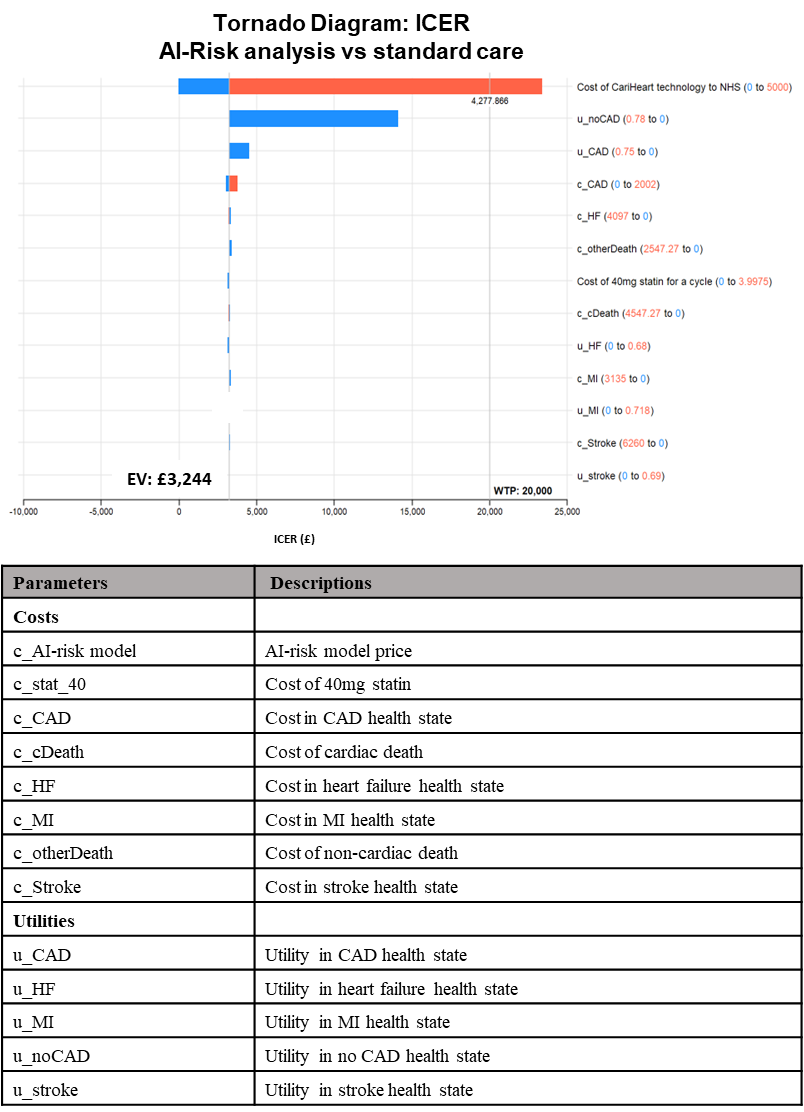
**Supplementary Figure S6:** Tornado diagram with parameters of cost and utility on incremental cost effectiveness ratio (ICER). After removing each model parameter, the uncertainty around the expected value (EV) of £3,244 were all well under the willingness to pay (WTP) threshold of £20,000. The price of AI-Risk analysis is £4,278 when the incremental cost effectiveness ratio (ICER) is at WTP threshold (Top bar).

**
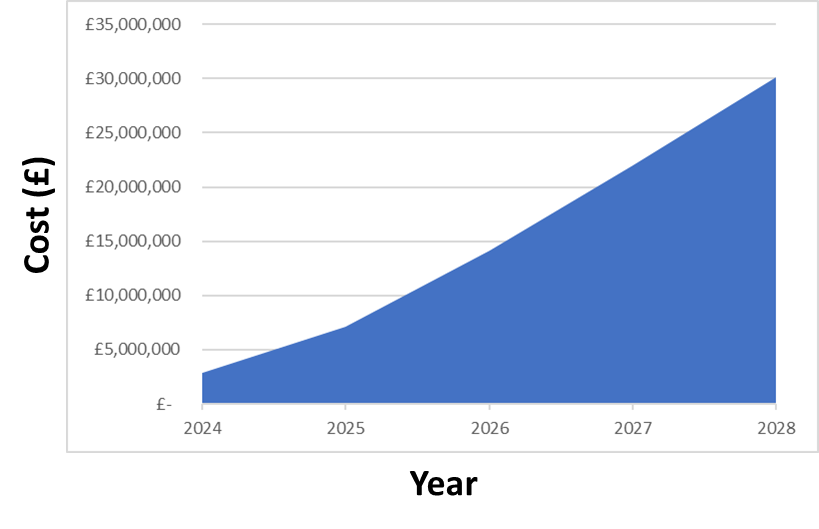
**

**Supplementary figure S7.** Budget impact analysis for deployment of the AI-Risk classification in the UK National Health System. The budget impact was estimated over 5 years and the annual uptake of AI-Risk model in the NHS was assumed to be 2%, 5%, 10%, 15%, and 20% in that period.

**
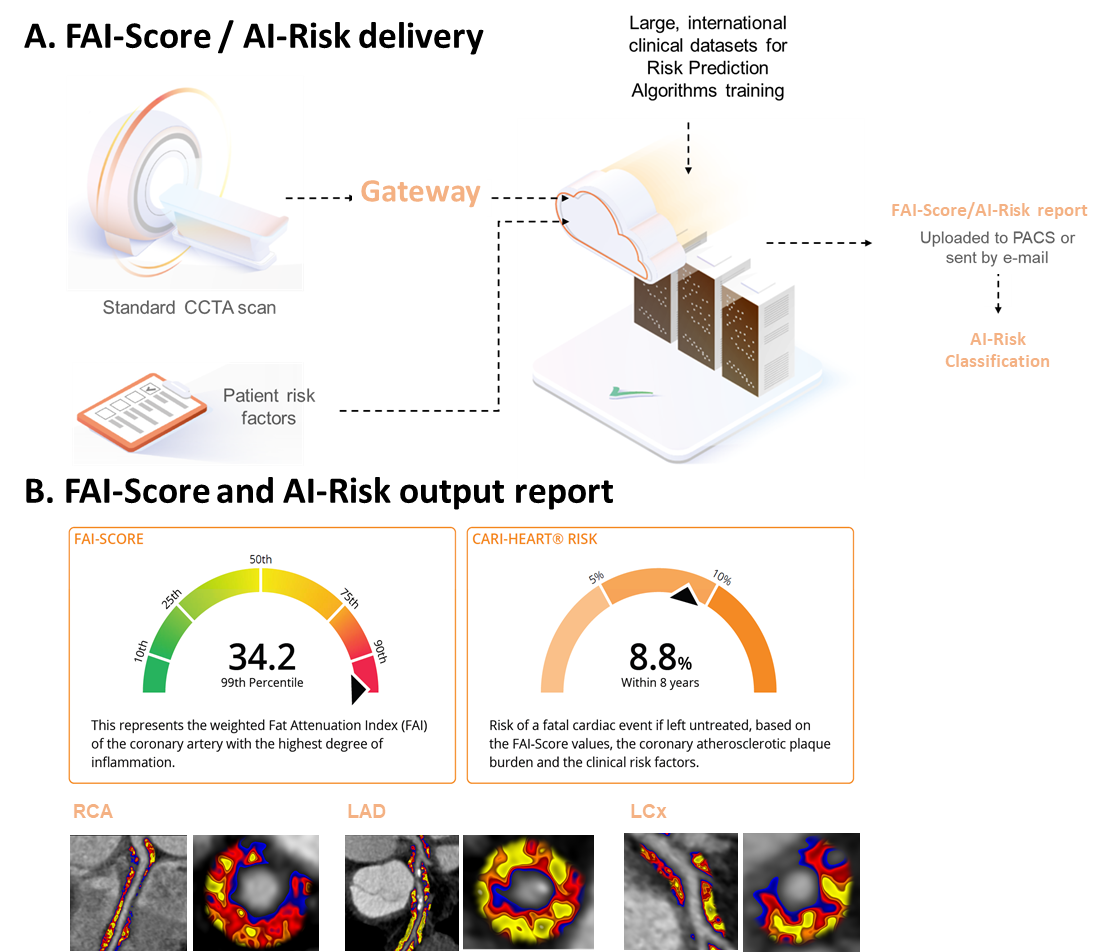
**

**Supplementary figure S8:** A) Overview of the software as a service (SAAS) system for delivery of FAI-Score and AI-Risk in clinical practice. B) Example output of FAI-Score and AI-Risk of a 56 year-old female with hyperlipidaemia. CT coronary angiography showed no significant luminal stenosis. FAI-Score for each coronary artery projected in risk nomograms for the age- and sex- to guide the interpretation of risk.

**Supplementary table S7.** Budget Impact Calculations for deployment of the AI-Risk classification

| **Budget Impact Calculations** | | |  |  |  |  |
| --- | --- | --- | --- | --- | --- | --- |
|  |  |  |  |  |  |  |
| **Year** | **Year 1** | **Year 2** | **Year 3** | **Year 4** | **Year 5** | **TOTAL** |
| **Population** | |  |  |  |  |  |
| **Current budget scenario - Standard of Care only** | | | |  |  |  |
| **Number of patients receiving CUA** | | | | | | |
| 2024 | 200,000 |  |  |  |  | 200,000 |
| 2025 | 200,000 | 200,000 |  |  |  | 400,000 |
| 2026 | 200,000 | 200,000 | 200,000 |  |  | 600,000 |
| 2027 | 200,000 | 200,000 | 200,000 | 200,000 |  | 800,000 |
| 2028 | 200,000 | 200,000 | 200,000 | 200,000 | 200,000 | 1,000,000 |
|  |  |  |  |  |  |  |
| **Year** | **Year 1** | **Year 2** | **Year 3** | **Year 4** | **Year 5** | **TOTAL** |
| **New budget scenario - gradual uptake of AI-risk model** | | | |  |  |  |
| **New Treatment uptake %** | 2% | 5% | 10% | 15% | 20% |  |
| **Patients receiving CUA** | |  |  |  |  |  |
| 2024 | 196,000 |  |  |  |  | 196000 |
| 2025 | 190,000 | 196,000 |  |  |  | 386000 |
| 2026 | 180,000 | 190,000 | 196,000 |  |  | 566000 |
| 2027 | 170,000 | 180,000 | 190,000 | 196,000 |  | 736000 |
| 2028 | 160,000 | 170,000 | 180,000 | 190,000 | 196,000 | 896000 |
| **Patients receiving New Treatment** | | |  |  |  |  |
| 2024 | 4,000 |  |  |  |  | 4000 |
| 2025 | 10,000 | 4,000 |  |  |  | 14000 |
| 2026 | 20,000 | 10,000 | 4,000 |  |  | 34000 |
| 2027 | 30,000 | 20,000 | 10,000 | 4,000 |  | 64,000 |
| 2028 | 40,000 | 30,000 | 20,000 | 10,000 | 4,000 | 104,000 |
|  |  |  |  |  |  |  |
| **Total Treatment Costs per Individual per Year** | | | | | | |
| **Cost per year** | **Year1** | **Year2** | **Year3** | **Year4** | **Year5** |  |
| CUA | £115.26 | £142.52 | £154.66 | £164.41 | £172.06 |  |
| AI-risk analysis | £820.75 | £147.95 | £159.28 | £168.25 | £175.16 |  |
|  |  |  |  |  |  |  |
| **Budget Impact Calculations** | | |  |  |  |  |
| **Current budget scenario - Standard of Care only** | | | |  |  | **ANNUAL TOTALS** |
| 2024 | 3,052,228 |  |  |  |  | 23,052,228 |
| 2025 | 3,052,228 | 8,504,221 |  |  |  | 51,556,450 |
| 2026 | 3,052,228 | 8,504,221 | 0,932,165 |  |  | 82,488,614 |
| 2027 | 3,052,228 | 8,504,221 | 0,932,165 | 2,882,229 |  | 15,370,843 |
| 2028 | 3,052,228 | 8,504,221 | 0,932,165 | 2,882,229 | 4,412,200 | 49,783,043 |
|  |  |  |  |  |  |  |
| **New budget scenario - gradual uptake of AI-risk model** | | | |  |  | **ANNUAL TOTALS** |
| **Cost for Patients receiving Standard of Care** | | | |  |  |  |
| 2024 | 2,591,184 |  |  |  |  | 22,591,184 |
| 2025 | 1,899,617 | 7,934,137 |  |  |  | 49,833,754 |
| 2026 | 0,747,006 | 7,079,010 | 0,313,521 |  |  | 78,139,537 |
| 2027 | 9,594,394 | 5,653,799 | 9,385,556 | 2,882,229 |  | 07,515,978 |
| 2028 | 8,441,783 | 4,228,588 | 7,838,948 | 2,882,229 | 3,723,956 | 37,115,503 |
| **Cost for Patients receiving New Treatment** | | | |  |  |  |
| 2024 | 3,283,015 |  |  |  |  | 3,283,015 |
| 2025 | 8,207,538 | 591,809 |  |  |  | 8,799,347 |
| 2026 | 6,415,076 | 1,479,523 | 637,127 |  |  | 18,531,725 |
| 2027 | 4,622,614 | 2,959,045 | 1,592,817 | 673,006 |  | 29,847,483 |
| 2028 | 2,830,152 | 4,438,568 | 3,185,635 | 1,682,516 | 700,624 | 42,837,494 |
| **Cost Sum for Patients receiving Standard of Care or New Treatment** | | | | | | |
| 2024 | 25,874,199 | - | - | - | - | 25,874,199 |
| 2025 | 30,107,155 | 28,525,946 | - | - | - | 58,633,101 |
| 2026 | 37,162,082 | 28,558,533 | 30,950,648 | - | - | 96,671,263 |
| 2027 | 44,217,008 | 28,612,844 | 30,978,374 | 33,555,235 | - | 137,363,461 |
| 2028 | 51,271,935 | 28,667,156 | 31,024,583 | 34,564,745 | 34,424,579 | 179,952,997 |
|  |  |  |  |  |  | - |
| **Net Budget Impact** | **Uptake** |  |  |  |  | **BUDGET IMPACT** |
| 2024 | 2% |  |  |  |  | £2,821,971 |
| 2025 | 5% |  |  |  |  | £7,076,651 |
| 2026 | 10% |  |  |  |  | £14,182,648 |
| 2027 | 15% |  |  |  |  | £21,992,618 |
| 2028 | 20% |  |  |  |  | £30,169,955 |

**Supplementary references:**

1. Chan K, Wahome E, Tsiachristas A*, et al.* Inflammatory risk and cardiovascular events in patients without obstructive coronary artery disease: the ORFAN multicentre, longitudinal cohort study. *Lancet* 2024;**403**:2606-2618. doi: 10.1016/S0140-6736(24)00596-8

2. Oikonomou EK, Antonopoulos AS, Schottlander D*, et al.* Standardized measurement of coronary inflammation using cardiovascular computed tomography: integration in clinical care as a prognostic medical device. *Cardiovasc Res* 2021;**117**:2677-2690. doi: 10.1093/cvr/cvab286

3. Chan K, Wahome E, Tsiachristas A*, et al.* Inflammatory risk and cardiovascular events in patients without obstructive coronary artery disease (The Oxford Risk Factors And Non-invasive imaging study): A multicentre, longitudinal cohort study. *under revision*. doi:

4. MHRA. Regulating medical devices in the UK. In: Agency MaHpR, (ed); 2023.

5. NICE. CVD risk assessment and management (CG181). In: National Institute for Health and Care Excellence; 2020.

6. NICE. Acute coronary syndromes NICE guideline (NG185). In: National Institute for Health and Care Excellence; 2020.

7. NICE. NICE Guidance for Stable Chest Pain Patients (CG95 & MTG32) to Appropriately Diagnose Patients with Suspected Coronary Artery Disease. In; 2019.

8. NICE. Cardiovascular disease: risk assessment and reduction, including lipid modification

NICE guideline [NG238]. In: National Institute for Health and Care Excellence; 2023.

9. Hippisley-Cox J, Coupland C, Brindle P. Development and validation of QRISK3 risk prediction algorithms to estimate future risk of cardiovascular disease: prospective cohort study. *BMJ* 2017;**357**:j2099. doi: 10.1136/bmj.j2099

10. Briggs AH, Gray AM. Power and sample size calculations for stochastic cost-effectiveness analysis. *Med Decis Making* 1998;**18**:S81-92. doi: 10.1177/0272989X98018002S10

11. Danese MD, Gleeson M, Kutikova L*, et al.* Estimating the economic burden of cardiovascular events in patients receiving lipid-modifying therapy in the UK. *BMJ Open* 2016;**6**:e011805. doi: 10.1136/bmjopen-2016-011805

12. Keng MJ, Leal J, Bowman L*, et al.* Hospital costs associated with adverse events in people with diabetes in the UK. *Diabetes Obes Metab* 2022;**24**:2108-2117. doi: 10.1111/dom.14796

13. Zhou J, Wu R, Williams C*, et al.* Prediction Models for Individual-Level Healthcare Costs Associated with Cardiovascular Events in the UK. *Pharmacoeconomics* 2023;**41**:547-559. doi: 10.1007/s40273-022-01219-6

14. Betts MB, Rane P, Bergrath E*, et al.* Utility value estimates in cardiovascular disease and the effect of changing elicitation methods: a systematic literature review. *Health Qual Life Outcomes* 2020;**18**:251. doi: 10.1186/s12955-020-01407-y

15. Kraai IH, Vermeulen KM, Luttik ML*, et al.* Preferences of heart failure patients in daily clinical practice: quality of life or longevity? *Eur J Heart Fail* 2013;**15**:1113-1121. doi: 10.1093/eurjhf/hft071

16. Sterne JA, Bodalia PN, Bryden PA*, et al.* Oral anticoagulants for primary prevention, treatment and secondary prevention of venous thromboembolic disease, and for prevention of stroke in atrial fibrillation: systematic review, network meta-analysis and cost-effectiveness analysis. *Health Technol Assess* 2017;**21**:1-386. doi: 10.3310/hta21090

17. Baigent C, Keech A, Kearney PM*, et al.* Efficacy and safety of cholesterol-lowering treatment: prospective meta-analysis of data from 90,056 participants in 14 randomised trials of statins. *Lancet* 2005;**366**:1267-1278. doi: 10.1016/S0140-6736(05)67394-1

18. Cholesterol Treatment Trialists C, Mihaylova B, Emberson J*, et al.* The effects of lowering LDL cholesterol with statin therapy in people at low risk of vascular disease: meta-analysis of individual data from 27 randomised trials. *Lancet* 2012;**380**:581-590. doi: 10.1016/S0140-6736(12)60367-5

**CHEERS 2022 Checklist for the health economic evaluation**

| **Topic** | **No.** | **Item** | **Location where item is reported** |
| --- | --- | --- | --- |
| **Title** |  |  |  |
|  | 1 | Identify the study as an economic evaluation and specify the interventions being compared. | **Page 1** |
| **Abstract** |  |  |  |
|  | 2 | Provide a structured summary that highlights context, key methods, results, and alternative analyses. | **Page 2** |
| **Introduction** |  |  |  |
| **Background and objectives** | 3 | Give the context for the study, the study question, and its practical relevance for decision making in policy or practice. | **Page 4-5** |
| **Methods** |  |  |  |
| **Health economic analysis plan** | 4 | Indicate whether a health economic analysis plan was developed and where available. | **Page 8-10** |
| **Study population** | 5 | Describe characteristics of the study population (such as age range, demographics, socioeconomic, or clinical characteristics). | **Page 5-8** |
| **Setting and location** | 6 | Provide relevant contextual information that may influence findings. | **Page 5-8** |
| **Comparators** | 7 | Describe the interventions or strategies being compared and why chosen. | **Page 8-10** |
| **Perspective** | 8 | State the perspective(s) adopted by the study and why chosen. | **Page 8-10** |
| **Time horizon** | 9 | State the time horizon for the study and why appropriate. | **Page 8** |
| **Discount rate** | 10 | Report the discount rate(s) and reason chosen. | **Page 9** |
| **Selection of outcomes** | 11 | Describe what outcomes were used as the measure(s) of benefit(s) and harm(s). | **Page 8** |
| **Measurement of outcomes** | 12 | Describe how outcomes used to capture benefit(s) and harm(s) were measured. | **Page 8** |
| **Valuation of outcomes** | 13 | Describe the population and methods used to measure and value outcomes. | **Page 8** |
| **Measurement and valuation of resources and costs** | 14 | Describe how costs were valued. | **supp materials page 19** |
| **Currency, price date, and conversion** | 15 | Report the dates of the estimated resource quantities and unit costs, plus the currency and year of conversion. | **supp materials page 19** |
| **Rationale and description of model** | 16 | If modelling is used, describe in detail and why used. Report if the model is publicly available and where it can be accessed. | **Page 8** |
| **Analytics and assumptions** | 17 | Describe any methods for analysing or statistically transforming data, any extrapolation methods, and approaches for validating any model used. | **Page 8** |
| **Characterising heterogeneity** | 18 | Describe any methods used for estimating how the results of the study vary for subgroups. | **Page 10** |
| **Characterising distributional effects** | 19 | Describe how impacts are distributed across different individuals or adjustments made to reflect priority populations. | **NA** |
| **Characterising uncertainty** | 20 | Describe methods to characterise any sources of uncertainty in the analysis. | **Page 10** |
| **Approach to engagement with patients and others affected by the study** | 21 | Describe any approaches to engage patients or service recipients, the general public, communities, or stakeholders (such as clinicians or payers) in the design of the study. | **Page 5** |
| **Results** |  |  |  |
| **Study parameters** | 22 | Report all analytic inputs (such as values, ranges, references) including uncertainty or distributional assumptions. | **Supp materials page 10-16** |
| **Summary of main results** | 23 | Report the mean values for the main categories of costs and outcomes of interest and summarise them in the most appropriate overall measure. | **Table 11** |
| **Effect of uncertainty** | 24 | Describe how uncertainty about analytic judgments, inputs, or projections affect findings. Report the effect of choice of discount rate and time horizon, if applicable. | **Table 2, supp figure S3-6** |
| **Effect of engagement with patients and others affected by the study** | 25 | Report on any difference patient/service recipient, general public, community, or stakeholder involvement made to the approach or findings of the study | **Page 5-6** |
| **Discussion** |  |  |  |
| **Study findings, limitations, generalisability, and current knowledge** | 26 | Report key findings, limitations, ethical or equity considerations not captured, and how these could affect patients, policy, or practice. | **Page 12-16** |
| **Other relevant information** |  |  |  |
| **Source of funding** | 27 | Describe how the study was funded and any role of the funder in the identification, design, conduct, and reporting of the analysis | **Page 16-17** |
| **Conflicts of interest** | 28 | Report authors conflicts of interest according to journal or International Committee of Medical Journal Editors requirements. | **Page 17** |

*From:* Husereau D, Drummond M, Augustovski F, et al. Consolidated Health Economic Evaluation Reporting Standards 2022 (CHEERS 2022) Explanation and Elaboration: A Report of the ISPOR CHEERS II Good Practices Task Force. Value Health 2022;25. <doi:10.1016/j.jval.2021.10.008>
